# PREDICT validity for prognosis of breast cancer patients with pathogenic *BRCA1/2* variants

**DOI:** 10.1038/s41523-023-00546-x

**Published:** 2023-05-12

**Authors:** Taru A. Muranen, Anna Morra, Sofia Khan, Daniel R. Barnes, Manjeet K. Bolla, Joe Dennis, Renske Keeman, Goska Leslie, Michael T. Parsons, Qin Wang, Thomas U. Ahearn, Kristiina Aittomäki, Irene L. Andrulis, Banu K. Arun, Sabine Behrens, Katarzyna Bialkowska, Stig E. Bojesen, Nicola J. Camp, Jenny Chang-Claude, Kamila Czene, Peter Devilee, HEBON investigators, Susan M. Domchek, Alison M. Dunning, Christoph Engel, D. Gareth Evans, Manuela Gago-Dominguez, Montserrat García-Closas, Anne-Marie Gerdes, Gord Glendon, Pascal Guénel, Eric Hahnen, Ute Hamann, Helen Hanson, Maartje J. Hooning, Reiner Hoppe, Louise Izatt, Anna Jakubowska, Paul A. James, Vessela N. Kristensen, Fiona Lalloo, Geoffrey J. Lindeman, Arto Mannermaa, Sara Margolin, Susan L. Neuhausen, William G. Newman, Paolo Peterlongo, Kelly-Anne Phillips, Miquel Angel Pujana, Johanna Rantala, Karina Rønlund, Emmanouil Saloustros, Rita K. Schmutzler, Andreas Schneeweiss, Christian F. Singer, Maija Suvanto, Yen Yen Tan, Manuel R. Teixeira, Mads Thomassen, Marc Tischkowitz, Vishakha Tripathi, Barbara Wappenschmidt, Emily Zhao, Douglas F. Easton, Antonis C. Antoniou, Georgia Chenevix-Trench, Paul D. P. Pharoah, Marjanka K. Schmidt, Carl Blomqvist, Heli Nevanlinna

**Affiliations:** 1 Department of Obstetrics and Gynecology, Helsinki University Hospital, University of Helsinki, Helsinki, Finland; 2 Research Program in Systems Oncology, Department of Biochemistry and Developmental Biology, University of Helsinki, Helsinki, Finland; 3 Division of Molecular Pathology, The Netherlands Cancer Institute, Amsterdam, the Netherlands; 4 Department of Genetics, HUSLAB, HUS Diagnostic Center, Helsinki University Hospital, University of Helsinki, Helsinki, Finland; 5 Individualized Drug Therapy Research Program, University of Helsinki, Helsinki, Finland; 6 Department of Clinical Pharmacology, University of Helsinki, Helsinki, Finland; 7 Centre for Cancer Genetic Epidemiology, Department of Public Health and Primary Care, University of Cambridge, Cambridge, UK; 8 Population Health Division, QIMR Berghofer Medical Research Institute, Brisbane, Queensland Australia; 9 Division of Cancer Epidemiology and Genetics, National Cancer Institute, National Institutes of Health, Department of Health and Human Services, Bethesda, MD USA; 10 Department of Medical and Clinical Genetics, University of Helsinki, Helsinki, Finland; 11 Fred A. Litwin Center for Cancer Genetics, Lunenfeld-Tanenbaum Research Institute of Mount Sinai Hospital, Toronto, Ontario, Canada; 12 Department of Molecular Genetics, University of Toronto, Toronto, Ontario, Canada; 13 Department of Breast Medical Oncology, University of Texas MD Anderson Cancer Center, Houston, TX USA; 14 Division of Cancer Epidemiology, German Cancer Research Center (DKFZ), Heidelberg, Germany; 15 Department of Genetics and Pathology, Pomeranian Medical University, Szczecin, Poland; 16 Copenhagen General Population Study, Herlev and Gentofte Hospital, Copenhagen University Hospital, Herlev, Denmark; 17 Department of Clinical Biochemistry, Herlev and Gentofte Hospital, Copenhagen University Hospital, Herlev, Denmark; 18 Faculty of Health and Medical Sciences, University of Copenhagen, Copenhagen, Denmark; 19 Department of Internal Medicine and Huntsman Cancer Institute, University of Utah, Salt Lake City, UT USA; 20 Cancer Epidemiology Group, University Cancer Center Hamburg (UCCH), University Medical Center Hamburg-Eppendorf, Hamburg, Germany; 21 Department of Medical Epidemiology and Biostatistics, Karolinska Institutet, Stockholm, Sweden; 22 Department of Pathology, Leiden University Medical Center, Leiden, the Netherlands; 23 Department of Human Genetics, Leiden University Medical Center, Leiden, the Netherlands; 24 Basser Center for BRCA, Abramson Cancer Center, University of Pennsylvania, Philadelphia, PA USA; 25 Centre for Cancer Genetic Epidemiology, Department of Oncology, University of Cambridge, Cambridge, UK; 26 Institute for Medical Informatics, Statistics and Epidemiology, University of Leipzig, Leipzig, Germany; 27 LIFE - Leipzig Research Centre for Civilization Diseases, University of Leipzig, Leipzig, Germany; 28 Prevent Breast Cancer Research Unit, The Nightingale Centre, Manchester University Hospital Foundation NHS Trust, Manchester, UK; 29 Division of Evolution and Genomic Sciences, School of Biological Sciences, Faculty of Biology, Medicine and Health, University of Manchester, Manchester Academic Health Science Centre, Manchester, UK; 30 Clinical Genetics Service, Manchester Centre for Genomic Medicine, Manchester University Hospitals Foundation Trust, Manchester, UK; 31 Manchester Breast Centre, Oglesby Cancer Research Centre, The Christie, University of Manchester, Manchester, UK; 32 Health Research Institute of Santiago de Compostela Foundation (FIDIS), SERGAS, Cancer Genetics and Epidemiology Group Santiago de Compostela, Santiago de Compostela, Spain; 33 Department of Clinical Genetics, Rigshospitalet, Copenhagen University Hospital, Copenhagen, Denmark; 34 Team “Exposome and Heredity”, CESP, Gustave Roussy, INSERM, University Paris-Saclay, UVSQ, Villejuif, France; 35 Center for Familial Breast and Ovarian Cancer, Faculty of Medicine and University Hospital Cologne, University of Cologne, Cologne, Germany; 36 Center for Integrated Oncology (CIO), Faculty of Medicine and University Hospital Cologne, University of Cologne, Cologne, Germany; 37 Molecular Genetics of Breast Cancer, German Cancer Research Center (DKFZ), Heidelberg, Germany; 38 SouthWest Thames Centre for Genomics, St George’s University Hospital’s NHS Foundation Trust, London, UK; 39 Department of Medical Oncology, Erasmus MC Cancer Institute, Rotterdam, the Netherlands; 40 Dr. Margarete Fischer-Bosch-Institute of Clinical Pharmacology, Stuttgart, Germany; 41 University of Tübingen, Tübingen, Germany; 42 Clinical Genetics, Guy’s and St Thomas’ NHS Foundation Trust, London, UK; 43 Independent Laboratory of Molecular Biology and Genetic Diagnostics, Pomeranian Medical University, Szczecin, Poland; 44 Parkville Familial Cancer Centre, The Royal Melbourne Hospital and Peter MacCallum Cancer Centre, Melbourne, Victoria, Australia; 45 Department of Medical Genetics, Oslo University Hospital and University of Oslo, Oslo, Norway; 46 Institute of Clinical Medicine, Faculty of Medicine, University of Oslo, Oslo, Norway; 47 Cancer Biology and Stem Cells Division, The Walter and Eliza Hall Institute of Medical Research, Parkville, Victoria, Australia; 48 Department of Medical Oncology, Peter MacCallum Cancer Center, Melbourne, Victoria, Australia; 49 Department of Medicine, Royal Melbourne Hospital, University of Melbourne, Parkville, Victoria, Australia; 50 Translational Cancer Research Area, University of Eastern Finland, Kuopio, Finland; 51 Institute of Clinical Medicine, Pathology and Forensic Medicine, University of Eastern Finland, Kuopio, Finland; 52 Biobank of Eastern Finland, Kuopio University Hospital, Kuopio, Finland; 53 Department of Oncology, Stockholm South General Hospital (Södersjukhuset), Stockholm, Sweden; 54 Department of Clinical Science and Education, Södersjukhuset, Karolinska Institutet, Stockholm, Sweden; 55 Department of Population Sciences, Beckman Research Institute of City of Hope, Duarte, CA USA; 56 North West Genomics Laboratory Hub, Manchester Centre for Genomic Medicine, St Mary’s Hospital, Manchester University NHS Foundation Trust, Manchester Academic Health Science Centre, Manchester, UK; 57 Genome Diagnostics Program, IFOM ETS - The AIRC Institute of Molecular Oncology, Milan, Italy; 58 Sir Peter MacCallum Department of Oncology, The University of Melbourne, Parkville, Victoria, Australia; 59 Department of Medicine, St Vincent’s Hospital, The University of Melbourne, Fitzroy, Victoria, Australia; 60 Centre for Epidemiology and Biostatistics, Melbourne School of Population and Global Health, The University of Melbourne, Melbourne, Victoria, Australia; 61 Translational Research Laboratory, IDIBELL (Bellvitge Biomedical Research Institute), Catalan Institute of Oncology, CIBERONC, Barcelona, Spain; 62 Clinical Genetics, Karolinska Institutet, Stockholm, Sweden; 63 Department of Clinical Genetics, University Hospital of Southern Denmark, Vejle Hospital, Vejle, Denmark; 64 Department of Oncology, University Hospital of Larissa, Larissa, Greece; 65 National Center for Tumor Diseases, University Hospital and German Cancer Research Center, Heidelberg, Germany; 66 Molecular Biology of Breast Cancer, University Womens Clinic Heidelberg, University of Heidelberg, Heidelberg, Germany; 67 Dept of OB/GYN and Comprehensive Cancer Center, Medical University of Vienna, Vienna, Austria; 68 Department of Genetics, Portuguese Oncology Institute, Porto, Portugal; 69 School of Medicine and Biomedical Sciences (ICBAS), University of Porto, Porto, Portugal; 70 Department of Clinical Genetics, Odense University Hospital, Odence C, Denmark; 71 Department of Medical Genetics, National Institute for Health Research Cambridge Biomedical Research Centre, University of Cambridge, Cambridge, UK; 72 Cancer Division, QIMR Berghofer Medical Research Institute, Brisbane, Queensland Australia; 73 Division of Psychosocial Research and Epidemiology, The Netherlands Cancer Institute - Antoni van Leeuwenhoek Hospital, Amsterdam, the Netherlands; 74 Department of Clinical Genetics, Leiden University Medical Center, Leiden, the Netherlands; 75 Department of Oncology, Helsinki University Hospital, University of Helsinki, Helsinki, Finland

**Keywords:** Breast cancer, Prognostic markers, Cancer genetics, Chemotherapy

## Abstract

We assessed the PREDICT v 2.2 for prognosis of breast cancer patients with pathogenic germline *BRCA1* and *BRCA2* variants, using follow-up data from 5453 *BRCA1/2* carriers from the Consortium of Investigators of Modifiers of *BRCA1/2* (CIMBA) and the Breast Cancer Association Consortium (BCAC). PREDICT for estrogen receptor (ER)-negative breast cancer had modest discrimination for *BRCA1* carrier patients overall (Gönen & Heller unbiased concordance 0.65 in CIMBA, 0.64 in BCAC), but it distinguished clearly the high-mortality group from lower risk categories. In an analysis of low to high risk categories by PREDICT score percentiles, the observed mortality was consistently lower than the expected mortality, but the confidence intervals always included the calibration slope. Altogether, our results encourage the use of the PREDICT ER-negative model in management of breast cancer patients with germline *BRCA1* variants. For the PREDICT ER-positive model, the discrimination was slightly lower in *BRCA2* variant carriers (concordance 0.60 in CIMBA, 0.65 in BCAC). Especially, inclusion of the tumor grade distorted the prognostic estimates. The breast cancer mortality of *BRCA2* carriers was underestimated at the low end of the PREDICT score distribution, whereas at the high end, the mortality was overestimated. These data suggest that *BRCA2* status should also be taken into consideration with tumor characteristics, when estimating the prognosis of ER-positive breast cancer patients.

The online PREDICT tool for estimating breast cancer patient prognosis has been widely adopted by clinicians during the past decade^1,2^. The algorithm for expected mortality for up to 10 years after breast cancer diagnosis has been validated in patient cohorts from Western Europe, North America, and South-East Asia^3–8^. PREDICT handles estrogen receptor (ER)-positive and ER-negative breast cancers as distinct disease entities^2^. In either case, PREDICT estimates the prognosis according to a baseline hazard function and a proportional prognostic score, based on diagnosis age and tumor characteristics, such as size and grade, ki67 and HER2 expression, and the number of affected lymph nodes. Furthermore, the progesterone receptor expression (PgR) will be incorporated in the score in the near future^9^. In addition to expected mortality, PREDICT estimates the absolute benefit from multiple treatment lines, including adjuvant endocrine therapy, 2nd or 3rd generation chemotherapy, trastuzumab, or bisphosphonates.

Pathogenic variants in *BRCA1* and *BRCA2* confer a high life-time risk of breast cancer and increased risk of ovarian cancer^10^. The *BRCA1* carrier breast tumors are characteristically triple-negative, high-grade carcinomas, whereas *BRCA2* carrier tumors are most often positive for estrogen receptor expression (ER-positive). The *BRCA1/2* carriers are diagnosed at a younger age when compared to non-carriers^11^, and the typical *BRCA1/2*-associated tumor characteristics are enriched in the younger age groups^12,13^. The overall survival rate of breast cancer patients with pathogenic *BRCA1/*2 variants is lower than the survival of non-carriers^14,15^. However, the difference may be largely explained by differences in tumor pathology and incidence of secondary ovarian cancer^16–18^. Intriguingly, some studies have suggested that the effects associated with the conventional pathological prognostic factors could be opposite in *BRCA1/2* carriers and non-carriers. For example, decreased survival of *BRCA1/2* carriers with ER-positive breast cancer has been reported in several studies^16,19–22^. Furthermore, the relevance of tumor grade as a prognostic factor for *BRCA1/2* has been questioned repeatedly^22,23^.

We have tested PREDICT model in retrospective follow-up data from *BRCA1/2* carrier patients from the Consortium of Investigators of Modifiers of *BRCA1/2* (CIMBA) and the Breast Cancer Association Consortium (BCAC).

## Results

Follow-up data was available for 2892 *BRCA1* and 1813 *BRCA2* variant carriers from CIMBA, and for 316 *BRCA1* and 432 *BRCA2* variant carriers from the BCAC. The pathology data was partially missing for many patients, but Multiple Imputation with Chained Equations (MICE, Supplementary Table 1) allowed inclusion of all patients with follow-up data. Multiple imputation requires that all statistical analyses are performed in parallel in the imputed datasets and that the analysis outputs are pooled for a final result. In the following, we use the term ‘pooled’ in this connotation. We performed all analyses separately for the ER-negative and ER-positive patient groups (Table 1), corresponding to the specific PREDICT models for ER-negative and ER-positive breast cancer and the characteristic tumor phenotypes of the *BRCA1* and *BRCA2* variant carriers, respectively. PREDICT scores and the expected breast cancer-associated mortality were calculated according to algorithm v 2.2., including variables for diagnosis age, tumor grade and size, lymph node and HER2 status, adjuvant therapy, and further adjusted for progesterone receptor expression^2,9,24,25^. The analyses included estimating the model discrimination, re-fitting the prognostic factors in a Cox regression model with the full score as an offset, and measuring the model calibration.

**Table 1:** Tumor characteristics of the *BRCA1/2* carrier breast cancer patients from CIMBA and BCAC in ER-specific subgroups.

	CIMBA				BCAC			
	*BRCA1* (ER-status known: 61.3%)		*BRCA2* (ER-status known: 68.4%)		*BRCA1* (ER-status known: 87.0%)		*BRCA2* (ER-status known: 83.8%)	
	ER− (75.7%)	ER+ (24.3%)	ER− (22.2%)	ER+ (77.8%)	ER− (71.6%)	ER+ (28.4%)	ER− (25.4%)	ER+ (74.6%)
PgR-negative	1140 (95.7%)	98 (27.3%)	193 (85%)	139 (17.9%)	172 (94.5%)	24 (36.4%)	70 (84.3%)	61 (27.5%)
PgR-positive	51 (4.3%)	261 (72.7%)	34 (15%)	637 (82.1%)	10 (5.5%)	42 (63.6%)	13 (15.7%)	161 (72.5%)
PgR status unknown	152 (11.3%)	71 (16.5%)	48 (17.5%)	189 (19.6%)	15 (7.6%)	12 (15.4%)	9 (9.8%)	48 (17.8%)
Her2-negative	793 (94.2%)	240 (86.6%)	146 (90.7%)	545 (88.8%)	127 (92%)	45 (91.8%)	53 (88.3%)	150 (83.8%)
Her2-positive	49 (5.8%)	37 (13.4%)	15 (9.3%)	69 (11.2%)	11 (8%)	4 (8.2%)	7 (11.7%)	29 (16.2%)
Her2 status unknown	501 (37.3%)	153 (35.6%)	114 (41.5%)	351 (36.4%)	59 (29.9%)	29 (37.2%)	32 (34.8%)	91 (33.7%)
Grade 1	9 (0.9%)	19 (5.6%)	6 (2.7%)	43 (5.6%)	1 (0.6%)	8 (12.5%)	1 (1.3%)	16 (6.6%)
Grade 2	123 (11.9%)	115 (33.7%)	43 (19%)	382 (49.6%)	26 (15.4%)	28 (43.8%)	19 (23.8%)	132 (54.5%)
Grade 3	902 (87.2%)	207 (60.7%)	177 (78.3%)	345 (44.8%)	142 (84%)	28 (43.8%)	60 (75%)	94 (38.8%)
Grade unknown	309 (23%)	89 (20.7%)	49 (17.8%)	195 (20.2%)	28 (14.2%)	14 (17.9%)	12 (13%)	28 (10.4%)
Size <=20 mm	604 (62.3%)	201 (63.2%)	124 (57.4%)	391 (55.7%)	95 (55.2%)	45 (65.2%)	39 (50%)	140 (59.3%)
Size >20 mm & <=50 mm	330 (34.1%)	110 (34.6%)	86 (39.8%)	271 (38.6%)	66 (38.4%)	22 (31.9%)	33 (42.3%)	83 (35.2%)
Size >50 mm	35 (3.6%)	7 (2.2%)	6 (2.8%)	40 (5.7%)	11 (6.4%)	2 (2.9%)	6 (7.7%)	13 (5.5%)
Size unknown	374 (27.8%)	112 (26%)	59 (21.5%)	263 (27.3%)	25 (12.7%)	9 (11.5%)	14 (15.2%)	34 (12.6%)
No affected lymph nodes	769 (70%)	226 (62.3%)	152 (64.4%)	397 (48.9%)	107 (61.1%)	44 (61.1%)	54 (67.5%)	116 (49.4%)
Any affected lymph nodes	329 (30%)	137 (37.7%)	84 (35.6%)	415 (51.1%)	68 (38.9%)	28 (38.9%)	26 (32.5%)	119 (50.6%)
Lymph node status unknown	245 (18.2%)	67 (15.6%)	39 (14.2%)	153 (15.9%)	22 (11.2%)	6 (7.7%)	12 (13%)	35 (13%)
No metastasis at diagnosis	93 (95.9%)	30 (100%)	29 (100%)	76 (96.2%)	75 (93.8%)	31 (93.9%)	45 (95.7%)	132 (97.1%)
Metastasis at diagnosis	4 (4.1%)	0 (0%)	0 (0%)	3 (3.8%)	5 (6.2%)	2 (6.1%)	2 (4.3%)	4 (2.9%)
Metastasis at dg unknown	1246 (92.8%)	400 (93%)	246 (89.5%)	886 (91.8%)	117 (59.4%)	45 (57.7%)	45 (48.9%)	134 (49,6%)

### ER-negative PREDICT

**Fig. 1:**
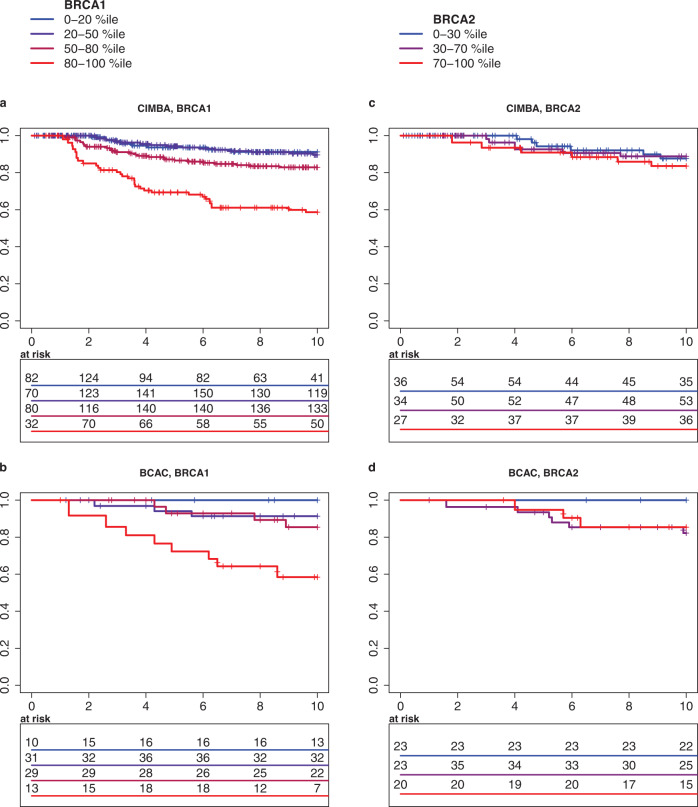
Survival of patients at distinct ER-negative PREDICT score percentiles. Kaplan–Meier survival estimator curves of *BRCA1* carriers with ER-negative breast cancer from those **a** CIMBA, **b** BCAC studies, which provided known cause of death for at least 80% of deceased patients. Similar visualization of the *BRCA2* carriers with ER-negative breast cancer from **c** CIMBA, **d** BCAC.

The ER-negative PREDICT score was able to discriminate the better and worse surviving *BRCA1* carriers with ER-negative breast cancer. In a study-stratified analysis of the CIMBA *BRCA1* carriers, the Gönen & Heller unbiased concordance for the PREDICT score with 15-year follow-up was 0.647, whereas in the analysis of the BCAC *BRCA1* carriers the concordance was 0.637 and in the analysis of the CIMBA *BRCA2* carriers 0.568. However, the model discrimination was slightly better when follow-up was restricted to the first five or ten years after the diagnosis (Table 2). The Gönen & Heller unbiased concordance derives the concordance probability directly from the Cox regression model. It is not dependent on uninterrupted follow-up and is therefore more reliable than AUC statistic for estimating discrimination in censored survival data. A concordance value of 0.50 suggests that a model is as good as a random guess and value 1.0 implies perfect prediction. The Kaplan–Meier graphs of patient survival at discrete risk levels provide visual evidence on the discriminatory potential of PREDICT for ER-negative breast cancer in *BRCA1* carriers (Fig. 1).

**Table 2:** Concordance of the PREDICT model for 5-, 10-, and 15-year follow-up for patients with ER-negative breast cancer.

Patient group	5-year concordance	10-year concordance	15-year concordance
CIMBA *BRCA1*	0.656 (0.648–0.663)^a^	0.651 (0.643–0.657)	0.647 (0.638–0.652)
CIMBA *BRCA2*	0.558 (0.523–0.576)	0.554 (0.536–0.568)	0.568 (0.549–0.590)
BCAC *BRCA1*	0.656 (0.646–0.668)	0.651 (0.641–0.662)	0.637 (0.626–0.650)

^a^Interquartile range of concordance estimates from imputed datasets in parenthesis.

We found no significant residual hazard associated with any of the tumor characteristics, on top of the ER-negative PREDICT score (Supplementary Table 2). Furthermore, a graphical examination of a spline of age-related hazard in the CIMBA *BRCA1* carriers suggested that the age-factor in the ER-negative PREDICT model fits well with the observed survival data (Supplementary Fig. 1).

**Fig. 2:**
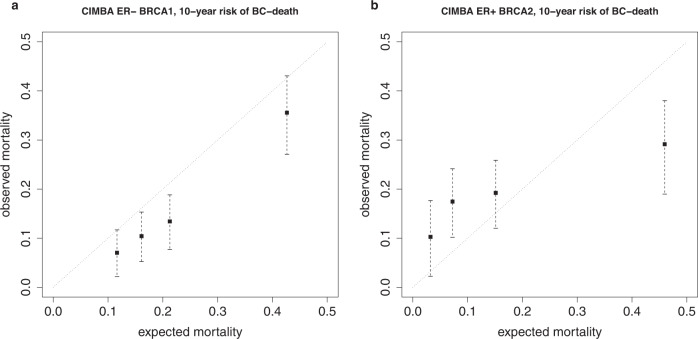
PREDICT calibration in CIMBA data. Point estimates and pooled 95% confidence intervals of observed mortality against expected mortality for **a** *BRCA1* carriers with ER-negative breast cancer, and **b** *BRCA2* carriers with ER-positive breast cancer.

The ER-negative PREDICT-algorithm overestimated breast cancer mortality in all *BRCA1/2* patient groups with ER-negative breast cancer from CIMBA and BCAC (Table 3). The pooled expected mortality was outside the 95% confidence interval of the pooled observed mortality when examining either all *BRCA1* or all *BRCA2* patients together (Table 3, first and two last rows). Consistently, when calibration was tested in CIMBA patient subgroups dichotomized by tumor size, grade, HER2 expression, node status, or in three distinct age categories, the expected mortality was higher than the observed mortality (Table 3). A calibration plot of low-to-high PREDICT percentiles in the *BRCA1* carriers with ER-negative breast cancer suggested a mild but consistent overestimation of 10-year mortality, with good separation of the middle-high (50–80%ile) and high (80–100%ile) mortality categories from middle-low (20–50%ile) and low categories (0–20%ile) (Fig. 2a). However, the difference between expected and observed mortality was slightly alleviated with a longer, 15-year, follow-up time (Supplementary Fig. 2).

**Table 3:** ER-negative PREDICT calibration measured by comparing the expected and observed breast 10-year cancer-specific mortality in patient groups with ER-negative breast cancer.

Patient group	Expected BC mortality	Observed BC mortality	95% CI of observed BC mortality
CIMBA *BRCA1*	0.23	0.16	0.13–0.19
Node-negative *BRCA1*^a^	0.18	0.12	0.08–0.15
Node-positive *BRCA1*^a^	0.50	0.45	0.33–0.55
Grade 2 *BRCA1*^a,b^	0.21	0.16	0.07–0.24
Grade 3 *BRCA1*^a,b^	0.23	0.16	0.13–0.19
Tumor size <= 20 mm *BRCA1*^a^	0.18	0.11	0.07–0.14
Tumor size >20 mm *BRCA1*^a^	0.30	0.24	0.18–0.29
HER2-negative^a^	0.22	0.16	0.13–0.20
HER2-positive^a^	0.27	0.11	0.02–0.19
Younger than 35 years *BRCA1*^a^	0.22	0.13	0.08–0.18
35–44 years old *BRCA1*^a^	0.23	0.16	0.12–0.20
45 years old or older *BRCA1*^a^	0.24	0.17	0.12–0.22
CIMBA *BRCA2*	0.25	0.15	0.08–0.21
BCAC *BRCA1*	0.28	0.21	0.14–0.27

^a^Subgroups of CIMBA BRCA1 patients with ER-negative breast cancer.

^b^Due to low number of patients with grade 1 breast cancer (see Table 1), this subgroup was not separately analyzed for calibration.

In summary, the PREDICT score predicted survival with modest precision in the ER-negative *BRCA1* carrier patients, although it tended to overestimate mortality throughout all risk levels. The prognostic impact of the individual risk factors in the PREDICT model did not deviate significantly from those of the PREDICT algorithm and the high-risk patients were identified well. Thus, the PREDICT model estimated the mortality risk in ER-negative *BRCA1* carriers with moderate accuracy, whereas for ER-negative *BRCA2*-carriers the analysis was indecisive, due to small cohort size.

### ER-positive PREDICT score

**Fig. 3:**
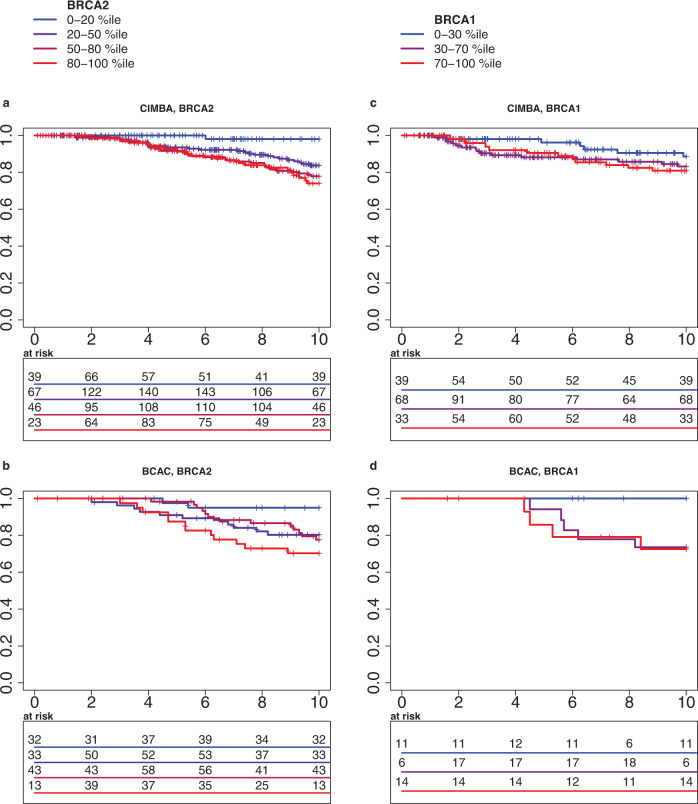
Survival of patients at distinct ER-positive PREDICT score percentiles. Kaplan–Meier survival estimator curves of *BRCA2* carriers with ER-positive breast cancer from merged **a** CIMBA, **b** BCAC studies, which provided known cause of death for at least 80% of deceased patients. *BRCA1* carriers with ER-positive breast cancer from **c** CIMBA, **d** from BCAC.

The ability of the PREDICT ER-positive model to discriminate *BRCA1/2* carriers was quite low in the CIMBA data, with Gönen & Heller concordance 0.601 for *BRCA2* carriers and 0.551 for *BRCA1* carriers, for follow-up time of 15 years after diagnosis, and equally poor for shorter follow-up of 10 years (Table 4). This was evident also in a modest separation of the Kaplan–Meier curves of *BRCA1/2* carriers with ER-positive breast cancer in different PREDICT percentile-based risk categories (Fig. 3). However, in the smaller dataset of *BRCA2* carriers from BCAC, the discrimination was higher, ranging from 0.665 for 5-year follow-up to 0.648 for 15-year follow-up (Table 4).

**Table 4:** Concordance of the PREDICT model for 5-, 10-, and 15-year follow-up for patients with ER-positive breast cancer.

Patient group	5-year concordance		10-year concordance		15-year concordance	
	ER+ PREDICT	reduced model^a^	ER+ PREDICT	reduced model	ER+ PREDICT	reduced model
CIMBA *BRCA2*	0.577 (0.560–0.589)^b^	0.587 (0.574–0.596)	0.604 (0.596–0.613)	0.615 (0.608–0.622)	0.601 (0.592–0.610)	0.610 (0.603–0.616)
CIMBA *BRCA1*	0.620 (0.587–0.643)	0.593 (0.573–0.618)	0.565 (0.533–0.580)	0.564 (0.539–0.581)	0.551 (0.531–0.570)	0.556 (0.536–0.573)
BCAC *BRCA2*	0.665 (0.655–0.674)	0.657 (0.648–0.664)	0.653 (0.649–0.661)	0.657 (0.649–0.664)	0.648 (0.642–0.652)	0.658 (0.650–0.662)

^a^Reduced model includes all factors of the ER + PREDICT model, except the tumor grade.

^b^Interquartile range of concordance estimates from imputed datasets in parenthesis.

When the pathologic factors, included in the PREDICT score, were refitted in a Cox regression model with the PREDICT score as an offset, to explain the survival of the CIMBA *BRCA2* carriers, the tumor grade had significant residual hazard in opposite direction to the coefficients embedded in the PREDICT (Table 5). A marginal residual hazard to opposite direction was seen also for PgR status, tumor size, and the number of affected lymph nodes, suggesting an overall poor fit of the PREDICT ER-positive score for the *BRCA2* carriers from CIMBA. When grade was removed from the PREDICT score, and included as an independent categorical covariate in a Cox regression model, offsetting with the reduced score, no significant difference was associated with either grade 3 or grade 1 when compared to grade 2 (Supplementary Table 3). Consequently, excluding grade from the PREDICT score improved the score concordance in CIMBA *BRCA2* carriers from 0.601 to 0.610, but also in BCAC *BRCA2* carriers from 0.648 to 0.658, suggesting that the tumor grade have little value in the prognosis of *BRCA2* carriers. A similar trend was seen also when restricting the follow-up time to ten years after diagnosis (Table 4).

**Table 5:** Residual hazard associated with PREDICT covariates.

Refitted factors	HR	95% CI	*P*
Diagnosis age (years)	1.01	0.99–1.03	0.27
Diagnosis year (1990–2011)	0.98	0.94–1.02	0.37
Tumor grade	0.42	0.32–0.56	1.8e-06
Progesterone receptor expression	1.75	1.12–2.75	0.04
HER2 expression	0.65	0.34–1.25	0.29
Tumor size (mm, log-transformed)	0.71	0.54–0.93	0.046
Positive lymph node count	0.96	0.93–1.00	0.096

All covariates were refitted in a country-stratified multivariable Cox regression model, offsetting with the ER-positive PREDICT score. The model was fitted to data from CIMBA *BRCA2* carriers with ER-positive breast cancer.

The PREDICT ER-positive score includes a non-linear component for diagnosis age, with steeply increasing hazard for ages younger than 40 years, and moderately increasing hazard for ages above 50 years. The relative hazard associated with diagnosis age had a milder curve in the CIMBA *BRCA2* carriers, when modeled with a spline. However, the PREDICT estimate was within the 95% confidence interval of the spline across ages 20 to 70 years (Supplementary Fig. 3).

The overall 10-year observed mortality of the *BRCA1/2* carriers with ER-positive breast cancer did not differ significantly from the PREDICT point estimate of expected mortality, either in data from CIMBA or BCAC (Table 6). However, a calibration plot of low to high risk categories of PREDICT percentiles (0–20%ile, 20–50%ile, 50–80%ile, 80–100%ile), suggested that PREDICT underestimated *BRCA2* carrier 10-year mortality in the lower risk categories, whereas in the high risk category, the observed mortality was significantly lower than the expected mortality (Fig. 2b). A longer follow-up time of 15-years did not affect the pattern (Supplementary Fig. 4). When examined in subgroups dichotomized by tumor pathology, the patients with grade 3 or node-positive tumors had lower mortality than expected, but patients with either grade 2 or node-negative tumors had higher mortality than expected (Table 6).

**Table 6:** ER-positive PREDICT calibration measured by comparing the expected and observed 10-year breast cancer-specific mortality in patient groups with ER-positive breast cancer.

Patient group	Expected BC mortality	Observed BC mortality	95% CI of observed BC mortality
CIMBA *BRCA2*	0.18	0.19	0.15–0.23
Node-negative *BRCA2*^a^	0.10	0.16	0.12–0.20
Node-positive *BRCA2*^a^	0.33	0.25	0.19–0.31
Grade 2 *BRCA2*^a,b^	0.12	0.20	0.15–0.25
Grade 3 *BRCA2*^a,b^	0.27	0.18	0.13–0.24
Tumor size <= 20 mm *BRCA2*^a^	0.10	0.13	0.08–0.17
Tumor size >20 mm *BRCA2*^a^	0.29	0.28	0.21–0.34
PgR-negative *BRCA2*^a^	0.25	0.16	0.06–0.24
PgR-positive *BRCA2*^a^	0.17	0.20	0.16–0.24
HER2-negative *BRCA2*^a^	0.18	0.20	0.16–0.23
HER2-positive *BRCA2*^a^	0.23	0.14	0.02–0.25
Younger than 35 years *BRCA2*^a^	0.31	0.19	0.10–0.28
35–44 years old *BRCA2*^a^	0.17	0.21	0.15–0.27
45 years old or older *BRCA2*^a^	0.15	0.18	0.13–0.22
CIMBA *BRCA1*	0.17	0.13	0.08–0.18
BCAC *BRCA2*	0.20	0.22	0.16–0.27

^a^Subgroups of CIMBA BRCA2 patients with ER-positive breast cancer.

^b^Due to low number of patients with grade 1 breast cancer (Table 1), this subgroup was not separately analyzed for calibration.

In summary, the accuracy of the PREDICT score for estimating the survival in ER-positive patients was lower than the accuracy in the ER-negative population. Although the PREDICT model estimated the average survival in the whole ER-positive patient population with moderate accuracy, the model did not reliably discriminate the low- and high-risk groups. Especially, the prognostic impact of the tumor grade deviated highly significantly from the PREDICT model, possibly reflecting underlying differences in the impact of tumor grade on prognosis in *BRCA1/2* carriers when compared to the patient populations on which the PREDICT model is based. In fact, the survival of *BRCA2* carriers with grade 3 tumors was similar to survival of *BRCA2* carriers with grade 2 tumors. Thus, the accuracy of the PREDICT model for estimating mortality risk in ER-positive *BRCA1*- or *BRCA2*-carriers was sub-optimal.

## Discussion

The primary motivation of PREDICT has been to provide a tool for clinicians to numerically estimate the benefit from adjuvant therapy. The relative benefit from adjuvant therapy is similar at all risk levels, but the absolute benefit is higher for patients at high risk of recurrence or cancer-associated death, making the risk of adverse side effects more acceptable in this group^2^. The algorithm was trained on a prospective population-based cohort from the UK, but multiple validation studies indicate that PREDICT gives reliable estimates also in many other populations^6,8,26^, despite significant differences in the baseline survival rates between countries^27^. Our analyses suggest, that the PREDICT ER-negative model is equally valid for management of *BRCA1/2* variant carriers with ER-negative breast cancer, but sub-optimal for estimating the prognosis of ER-positive breast cancer.

Previous validation analyses of PREDICT version 2 have measured the discrimination with AUC (area under curve) -statistics, ranging from 0.696 to 0.75 for the ER-negative model^2,8^. The concordance in the *BRCA1* carrier data was lower: 0.65 in data from CIMBA and 0.64 in data from BCAC, but sufficient to discriminate especially the poor survival group of the *BRCA1* patients (Fig. 2).

Despite the good discrimination, PREDICT seemed to overestimate the risk of breast cancer-specific death. The difference between expected and observed mortality was about 7–8 percentage points ten years after diagnosis, but decreased with a longer follow-up time of 15 years (Table 3, Fig. 2a, Supplementary Fig. 2). Van Maaren et al. previously reasoned that a difference of this magnitude has clinical impact, because it is sufficiently large to affect the treatment choice, whether to administer adjuvant chemotherapy^8^. However, over-estimating mortality is less detrimental than underestimating, because it does not risk the access to a sufficiently efficient adjuvant therapy. Of the CIMBA *BRCA1* carrier patients with ER-negative breast cancer, who had adjuvant therapy recorded in the data (none/any), about 90% had received adjuvant chemotherapy, even at the lowest risk category (PREDICT 0–20%ile). A beneficial treatment response is one possible explanation for the difference between expected and observed mortality, even though the expected benefit from adjuvant therapy was embedded in the PREDICT score. The difference may also have arisen from the imputation process. M-status was missing for a substantial number of patients (Table 1). Filtering the patients with imputed M-status may have caused loss of early events. However, the expected-observed difference was equally large in BCAC, where the M-status was more frequently available. Thus, this does not appear as a major source of bias, though it warrants caution in interpretation. Furthermore, the expected-observed difference is in keeping with a recent study, where the survival of *BRCA1* carriers breast cancer was nominally higher than survival of non-carriers in pathology- and treatment-adjusted analysis of patients with ER-negative breast cancer^18^.

*BRCA2* variant carrier cancers are characteristically ER-positive. However, a recent study suggested that germline *BRCA2* variants increase also the risk of triple-negative breast cancer, which is generally considered a poor-prognosis breast cancer subtype^13^. Our analyses on PREDICT in *BRCA2* carriers with ER-negative breast cancer were indecisive. The discrimination was low (0.568, Table 2), and breast cancer-associated survival good, with lower than expected mortality, similarly to the *BRCA1* carriers with ER-negative breast cancer (Table 3, Fig. 1).

In previous studies, validating the PREDICT model in cohorts of unselected breast cancer patients, the discrimination of the ER-positive model has consistently been higher than the discrimination of the ER-negative model, with AUC-statistics between 0.74 and 0.79^2,8^. In that respect, the PREDICT concordance of 0.60 in the CIMBA *BRCA2* carriers with ER-positive breast cancer appeared strikingly low.

The offset- and the calibration-analyses indicated that especially the tumor grade appeared to confuse the PREDICT ER-positive score, when predicting the *BRCA2* variant carrier survival, whereas the factors related to the stage of malignant progression, like tumor size and node involvement, retained their predictive potential. These observations were made in the CIMBA data, but as omitting grade from the score improved its discrimination also in the BCAC data, we can conclude that the same trend is present also there. Earlier studies have suggested that the survival of *BRCA2* carrier patients does not vary by tumor grade, after other pathologic factors have been taken into account^21–23^. In our analysis, where tumor grade was an independent covariate, the hazard associated with grade 3 in comparison to grade 2 was nominally lower, with a pooled *P*-value close to the significance threshold. However, in this kind of retrospective data, the observed survival differences cannot be separated from the treatment choice. Grade 3 *BRCA2* carriers had received more often adjuvant chemotherapy or combined chemo-endocrine therapy than grade 2 patients (Supplementary Table 4), and the underlying differences in therapeutic practices for grade 2 and grade 3 ER-positive cancers may have contributed to the nominally lower survival of the grade 2 patients.

The overall calibration of the PREDICT in *BRCA2* carriers with ER-positive breast cancer was good. However, the calibration varied by the magnitude of the PREDICT score, the observed mortality being higher than the expected, especially in the lower-risk groups (Table 6, Fig. 2b). A recent BCAC study, comparing the *BRCA1/2* carrier survival to survival of population matched non-carriers, found *BRCA2* pathogenic variants to be associated with decreased patient survival after ER-positive breast cancer^18^. Our analyses suggest that this difference would be emphasized in patient groups with milder clinical characteristics. Therefore, the PREDICT model does not appear well-suited for the management of *BRCA2* carriers with ER-positive breast cancer. Similarly, the concordance of 0.55 does not provide much support for the PREDICT model in the management of *BRCA1* variant carriers with ER-positive breast cancer, either.

As the purpose of the PREDICT is to aid in the decision on adjuvant therapy, the fundamental question in our study was, whether the *BRCA1/2* carriers could be managed the same way as non-carriers, and especially, is the *BRCA1/2* carrier status such vital information, that genotyping the patients prior to therapy would be advisable. Strong family history of breast and ovarian cancer indicates high likelihood of germline *BRCA1* or *BRCA2* pathogenic variant. However, genotyping to explore the causes of the familial risk may take place only after the management of the proband’s primary cancer. Furthermore, not all carriers have such family structure or records that would reveal the increased hereditary risk. Therefore, it’s likely that many variant carriers with breast cancer are treated without knowledge about the carrier status. The characteristic mutational signature of the *BRCA1/2* variant carrier cancer, homologous recombination deficiency^28^, makes the cancers responsive to platinum-based therapy or PARP-inhibitors^29,30^, but most of the carriers are still treated according to standard indications^31^. Retrospective analyses have suggested that the benefit from the standard adjuvant chemotherapy regimens are similar for *BRCA1/2* carriers and non-carriers, but the benefit from adjuvant endocrine therapy is limited^32,33^. Instead, oophorectomy has recently been suggested to reduce breast cancer recurrence and mortality of both *BRCA1* and *BRCA2* variant carriers^32–34^. In our study, the breast cancer-associated mortality of *BRCA2* carriers with ER-positive breast cancer was higher than expected in a patient group, where adjuvant chemotherapy was less-frequently used, but lower than expected in the high-risk patient group where adjuvant chemotherapy was used more often (Supplementary Table 4).

The strengths of this study include a large number of cases with pathogenic *BRCA1/2* germline variants and a stratified analysis of multiple cohorts from Europe, Northern America, and Australia. The study limitations include late recruitment of some patients and notable proportion of missing pathology and treatment data. To alleviate these shortcomings, the collected data has been harmonized and curated. Especially, we ensured that the number of patients under observations right after diagnosis was sufficiently high for an unbiased survival analysis. Furthermore, we applied statistical methods, like multiple imputation and left truncation to achieve robust conclusions. However, we were not able to address all nuances related to breast cancer diagnosis and management, like the presence of micrometastases, the duration of endocrine therapy, or administration of neoadjuvant chemotherapy. It’s worth noting that the PREDICT was not trained with a cohort that would have included patients treated with neoadjuvant therapy. We run a sensitivity analysis to exclude cases with known or imputed neoadjuvant chemotherapy. The results were essentially similar to the results of the main analyses, supporting the conclusions presented above.

The PREDICT ER-negative model gives reliable estimates, but the ER-positive model is less well-suited for *BRCA1/2* carriers. Especially, our analyses indicate *BRCA2* carriers a specific group of breast cancer patients, for whom the conventional prognostic estimation is not well-suited. Altogether, our findings encourage including the information on germ-line pathogenic *BRCA1/2* variants into the decision making for adjuvant therapy regimens of breast cancer patients.

## Methods

### Study subjects

The study subjects included female breast cancer patients of European ethnic origin enrolled into studies participating in the CIMBA (Table 7). For these analyses, the *BRCA1/2* carrier patients were considered eligible, if they were diagnosed with primary breast cancer under the age of 70 years, at 1990 or later, and had the following data available: follow-up time after the first invasive breast cancer diagnosis, status (dead/alive) at the end of follow-up, time of DNA sample collection, diagnosis age, and diagnosis year. Study subjects with previous ovarian cancer diagnosis or those included in the BCAC studies (see below) were excluded from CIMBA. This yielded data from 2892 *BRCA1*, 1813 *BRCA2* pathogenic variant carriers with breast cancer. The number of patients under observation right after diagnosis was 836, reached maximum, 2066, at about 4 years after diagnosis, steadily decreasing to 800 under observation 15 years after diagnosis.

**Table 7:** Contributing studies.

Consortium	Country stratum	Study	Acronym	Country	Study design	Name of the institutional review board (IRB)	Informed consent policy	Study subjects		Pathology data to support imputation	
								*BRCA1*	*BRCA2*	*BRCA1/2*	Non-carriers
CIMBA	Australia	Kathleen Cuningham Consortium for Research into Familial Breast Cancer	KCONFAB	Australia	Cancer clinic-based study	Peter MacCallum Cancer Centre Ethics Committee	WIC	315	246	64	
		Victorian Familial Cancer Trials Group	VFCTG	Australia	Cancer clinic-based study	Peter MacCallum Cancer Centre Ethics Committee	IRB^a^	67		52	
	Austria	General Hospital Vienna	MUV	Austria	Cancer clinic-based study	Ethikkommission der Medizinischen Universität Wien	WIC	189	99	58	
	Denmark	Copenhagen Breast Cancer Study	CBCS	Denmark	Cancer clinic-based study	De Videnskabsetiske Komiteer I Region Hovedsladen	IRB^b^	109	68	24	
		Odense University Hospital	OUH	Denmark	Cancer clinic-based study	Den Videnskabsetiske Komité for Region Syddanmark	IRB^b^	169	135	41	
	Finland	Helsinki Breast Cancer Study	HEBCS	Finland	Cancer clinic-based study	Helsingin ja uudenmaan sairaanhoitopiiri (Helsinki University Central Hospital ethics committee)	WIC	48	56	13	
	Germany	German Familial Breast Group	GC-HBOC	Germany	Cancer clinic-based study	Ethik-Kommission der Medizinischen Fakultät der Universät zu Köln	WIC	398	231	1149	
	Netherlands	Hereditary Breast and Ovarian cancer study the Netherlands	HEBON	Netherlands	Cancer clinic-based study	Protocol Toetsingscommissie van het Nederlands Kanker Instituut/Antoni van Leeuwenhoek Ziekenhuis	WIC	274	103	38	
	North America	Ontario site of the Breast Cancer Family Registry/Ontario Cancer Genetics Network	BCFR-ON/OCGN	Canada	Cancer clinic- and population-based study	Mount Sinai Hospital Research Ethics Board	WIC		45	77	
		City of Hope Cancer Center	COH	USA	Cancer clinic-based study	City of Hope Institutional Review Board	WIC	94		6	
		Dana Farber Cancer Institute	DFCI	USA	Cancer clinic-based study	Dana Farber Cancer Institute Institutional Review Board	WIC	58	43	14	
		University of Pennsylvania	UPENN	USA	Cancer clinic-based study	University of Pennsylvania Institutional Review Board	WIC	101		145	
		University of Texas MD Anderson Cancer Center	UTMDACC	USA	Cancer clinic-based study	University of Texas MD Anderson Cancer Center Office of Protocol Research Institutional Review Board	WIC	61		38	
	Poland	International Hereditary Cancer Centre	IHCC	Poland	Cancer clinic-based study	Komisja Bioetyczna Pomorskiej Akademii Medycznej (Pomeranian Medical University Bioethics Committee)	WIC	244		10	
	Sweden	Swedish Breast Cancer Study	SWE-BRCA	Sweden	Cancer clinic-based study	Regionala Etikprövningsnämnden Stockholm	WIC	83		48	
	Iberia	Institut Català d’Oncologia	ICO	Spain	Cancer clinic-based study	Catalan Institute of Oncology Institutional Review Board	WIC	78	86	86	
		Portuguese Oncology Institute-Porto Breast Cancer Study	IPOBCS	Portugal	Cancer clinic-based study	Comissão de Ética para a Saúde (CES) - IPO-Porto EPE	WIC		81	43	
	UK	Victorian Familial Cancer Trials Group	EMBRACE	UK	Cancer clinic-based study	Anglia & Oxford MREC	WIC	604	620	232	
BCAC	Australia	Kathleen Cuningham Foundation Consortium for research into Familial Breast Cancer/Australian Ovarian Cancer Study	KCONFAB/AOCS	Australia and New Zealand	Clinic-based recruitment of familial breast cancer patients	kConFab: Peter MacCallum Cancer Centre Ethics Committee	WIC		9	2	1002
		Melbourne Collaborative Cohort Study	MCCS	Australia	Prospective cohort study: nested case-control study	The Cancer Council Victoria Human Research Ethics Committee	WIC	3	7	1	652
	Central Europe	Breast Cancer Study of the University of Heidelberg	BSUCH	Germany	Hospital-based cases	Ethikkommission Medizinische Fakultat Heidelberg, University of Heidelberg	WIC	5	5		176
		CECILE Breast Cancer Study	CECILE	France	Population-based case-control study	Comité Consultatif de Protection des Personnes dans la Recherche Biomédicale de Bicêtre (Le Kremlin-Bicêtre FR-94270)	WIC	7	12	3	833
		German Consortium for Hereditary Breast & Ovarian Cancer	GC-HBOC	Germany	Clinic-based case study and prospective cohort study	Ethik-Kommission der Medizinischen Fakultat der Universitat zu Koln	WIC		4	16	2099
		Gene Environment Interaction and Breast Cancer in Germany	GENICA	Germany	Population-based case-control study	Ethikkommission Rheinische Friedrich-Wilhels-Universität Bonn	WIC	9	15		691
		Genetic Epidemiology Study of Breast Cancer by Age 50	GESBC	Germany	Population-based study of women <50 years	Medizinische Fakultat Heidelberg Ethikkommission	WIC	23	17		510
		Mammary Carcinoma Risk Factor Investigation	MARIE	Germany	Population-based case-control study	Medizinische Fakultat Heidelberg Ethikkommission; Ethik-Kommission der Arztekammer Hamburg	WIC	9	27	1	1999
		Städtisches Klinikum Karlsruhe Deutsches Krebsforschungszentrum Study	SKKDKFZS	Germany	Hospital-based breast cancer cohort	Medizinische Fakultat Heidelberg Ethikkommission	WIC	12	16	1	700
	Denmark	Copenhagen General Population Study	CGPS	Denmark	Population-based case-control study	Kobenhavns Amt den Videnskabsetiske Komite (Scientific ethical committee, Copenhagen County)	WIC	34	24	4	1893
	Finland	Helsinki Breast Cancer Study	HEBCS	Finland	Hospital-based case-control study, plus additional familial cases	Helsingin ja uudenmaan sairaanhoitopiiri (Helsinki University Hospital Ethics Committee)	WIC	8	13	1	1499
		Kuopio Breast Cancer Project	KBCP	Finland	Population-based prospective clinical cohort	Pohjois-Savon Sairraanhoitopiirin Kuntayhtyma Tutkimuseettinen Toimikunta	WIC	3	3	5	404
	Netherlands	Amsterdam Breast Cancer Study	ABCS	Netherlands	Hospital-based consecutive cases	Leiden University Medical Center (LUMC) Commissie Medische Ethiek; Protocol Toetsingscommissie van Het Nederlands Kanker Instituut-Antoni van Leeuwenhoek Ziekenhuis	WIC	27	26	26	962
		Amsterdam Breast Cancer Study - Familial	ABCS-F	Netherlands	Clinical Genetic Center-based cases	Leiden University Medical Center (LUMC) Commissie Medische Ethiek; Protocol Toetsingscommissie van Het Nederlands Kanker Instituut-Antoni van Leeuwenhoek Ziekenhuis	WIC				119
		Rotterdam Breast Cancer Study	RBCS	Netherlands	Hospital-based case-control study, Rotterdam area	Medische Ethische Toetsings Commissie Erasmus Medisch Centrum	WIC	5	2		894
	North America	Ontario Familial Breast Cancer Registry	OFBCR	Canada	Population-based familial case-control study	Mount Sinai Hospital Research Ethics Board	WIC	1	3		354
		The Prostate, Lung, Colorectal and Ovarian (PLCO) Cancer Screening Trial	PLCO	USA	Prospective cohort study: nested case-control	National Cancer Institute Special Studies Institutional Review Board (NCI-SSIRB)	WIC	2	15	1	1188
		Utah Breast Cancer Study	UBCS	USA	Pedigrees including multiple cases; Hospital-based cases; Population-based cases	University of Utah Institutional Review Board	WIC	5	7	5	486
	Poland	NCI Polish Breast Cancer Study	PBCS	Poland	Population-based case-control study	National Cancer Institute Special Studies Institutional Review Board (NCI-SSIRB)	WIC	52	29	6	1460
	Scandinavia	Karolinska Breast Cancer Study	KARBAC	Sweden	Population and hospital-based cases	Regionala Etikprovningsnamnden i Stockholm (Regional Ethical Review Board in Stockholm)	WIC	2	4		285
		Karolinska Mammography Project for Risk Prediction of Breast Cancer - Cohort Study	KARMA	Sweden	Cohort study	Regionala Etikprovningsnamnden i Stockholm (Regional Ethical Review Board in Stockholm)	WIC	13	19	2	2425
		Norwegian Breast Cancer Study	NBCS	Norway	Hospital-based case-control study	Regionale Komitere for Medisinsk og Helsefaglig Forskningsetikk	WIC	1	3		424
		Singapore and Sweden Breast Cancer Study	SASBAC	Sweden	Population-based case-control study	Regionala Etikprovningsnamnden i Stockholm (Regional Ethical Review Board in Stockholm)	WIC	7	3		903
	Southern Europe	Breast Oncology Galicia Network	BREOGAN	Spain	Population-based case-control	Comité Autonómico de Ética de la Investigación de Galicia	WIC	8	10	4	489
		Crete Cancer Genetics Program	CCGP	Greece	Hospital-based case-control study	Epistimoniko Symvoulio (Scientific Council of the University General hospital of Heraklion)	WIC	8	2		387
		Milan Breast Cancer Study Group	MBCSG	Italy	Clinic-based familial/early onset breast cancer patients	Comitato Etico Indipendente della Fondazione IRCCS “Istituto Nazionale dei Tumori”	WIC		2		425
	UK	Predicting the Risk Of Cancer At Screening Study	PROCAS	UK	Population based study	NRES Committee North West - Greater Manchester Central	WIC		4	1	322
		Study of Epidemiology and Risk factors in Cancer Heredity	SEARCH	UK	Population-based case-control study	Multi Centre Research Ethics Committee (MREC)	WIC	72	151	47	9331

WIC: All participants gave a written informed consent; IRB: Informed consent was waived by the ethical committee.

^a^The majority of participants in VFCTG have prospectively signed to give specific consent but a group of mutation carriers were included in the group retrospectively (in 2012) with a waiver of consent approved by the HREC at Peter MacCallum Cancer Centre: HREC/12/PMCC/29_project 12/111.

^b^Informed consent is not necessary in Denmark for this kind of studies. The study has been approved by the regulatory and juridical authorities.

Separate validation was performed in an independent set of *BRCA1/2* variant carriers from the BCAC. The variant carrier status was confirmed in gene panel sequencing as a part of the BRIDGES project^35^. Patients with *BRCA1/2* pathogenic or likely pathogenic variants (class 4 and 5) were included in the analyses^36^. Variant classification was downloaded from ClinVar in June 2020. The BCAC data came from patients enrolled for their first invasive breast cancer and included 316 *BRCA1* and 432 *BRCA2* variant carriers (Table 7). The number of patients under observation right after diagnosis was 229, reached maximum, 538, at about 4 years after diagnosis, steadily decreasing to 155 under observation 15 years after diagnosis.

The study was compliant with the Helsinki declaration. All participating studies were approved by their appropriate institutional review boards (Table 7), following their national guidelines for informed consent. The details on study-wise informed consent policies are provided in Table 7.

### Phenotype data

All available pathology, treatment, and follow-up data were retrieved from the consortium databases (CIMBA database version 2016, BCAC database release 13). Since these data were incomplete, we applied Multiple Imputation by Chained Equations (MICE) for imputation of the missing values, so that we were able to calculate the PREDICT scores for all patients with available survival data. We assumed that due to the complex relations between the variables, a maximally large sample of observed data would provide the best foundation for imputation. Therefore, additional data from 2138 *BRCA1/2* carriers from CIMBA as well as from 126 *BRCA1/2* carriers and 32912 non-carriers (including BRCA1/2 variants of unknown significance) from BCAC were included to support the imputation process (Table 7). Data management and statistical analyses were performed with R environment for statistical computing, version 4.0.0^37^.

We imputed missing data into 50 parallel data matrices with R library *mice*^38^. The pathology, treatment, and follow-up data from CIMBA and BCAC were harmonized in terms of variable names, types, and coding, and then combined. A Nelson-Aalen estimate of cumulative hazard of overall and breast cancer-specific survival until the end of follow-up time was calculated for all patients with available follow-up time and used in the imputation process. The Nelson-Aalen estimator for breast cancer-specific survival was defined on the basis of studies, which provided the cause of death (BC/other) for at least 80% of deceased patients. The prediction matrix, defining the relations of the imputed features, was initiated by pairwise correlation between the features, defined as Spearman rank correlation >0.125, and further modified as follows. Mutual prediction was forced between ER-status and PR-status, as well as ER-status and tumor morphology. Diagnosis year was not allowed to predict HER2-status. The tumor size category was predicted by correlated features, but tumor size in mm (log-scale) was predicted only by the size category (Supplementary Table 1). Data was post-processed so that adjuvant therapy subtypes were not positive, if the main type (chemo- or endocrine therapy) was not positive. Trastuzumab treatment was not allowed before year 1997. The imputed data was checked by cross-tabulation, to assure that the relations between covariables and the *BRCA1/2* specific features were retained.

### PREDICT scores

The PREDICT scores were calculated according to the functions presented at the PREDICT website (https://breast.predict.nhs.uk/legal/algorithm, accessed 2021-09-17), including coefficients or functions for diagnosis age, tumor grade (1, 2, 3), tumor largest diameter in mm, positive lymph node count, HER2 status (dichotomous), and fixed coefficients for adjuvant chemo- and endocrine therapy^2,24,25^. Furthermore, the coefficients associated with positive progesterone expression was added as suggested in Grootes et al.^9^. Patients with positive M-status (metastasis at diagnosis) were excluded after multiple imputation, since PREDICT is not applicable for M1 patients. The M-status was missing for a very high proportion of patients (Table 1) and the least biased approach in the context of multiple imputation is to filter the data only after the imputation process. Ki67 data was not available, and the corresponding coefficients were excluded from the score. The expected breast cancer mortality was calculated based on the PREDICT scores and baseline risks for breast cancer and other cause mortality^3^.

### Statistical analysis

The analyses were performed in parallel in the 50 imputed datasets, and the final results, e.g., regression model coefficients, model-based predictions, concordance, and expected or observed mortality were pooled according to Rubin’s rules or as recommended for event history analysis in Marshall et al.^39,40^. The PREDICT risk categories, defined by PREDICT score percentiles were pooled by voting—the pooled category for a patient was the category, which the patient received most frequently in the 50 imputed datasets. Survival analyses were performed with R library *survival*^41,42^. The 15-year follow-up started at the first breast cancer diagnosis, and left-truncation was applied to account for delayed entry. Patients were censored at the end of follow-up, if lost from follow-up, or at non-breast-cancer-related death.

The PREDICT ER-negative and ER-positive scores were tested separately in the corresponding subgroups of *BRCA1* and *BRCA2* carrier patients from CIMBA and BCAC. First, the PREDICT score was tested as a linear covariate in a Cox regression model, using the model concordance as a measure of the model fit. The Gönen & Heller unbiased concordance was estimated using R library *CPE*^43^ and pooled to median. Second, the PREDICT score was used as an offset factor in a Cox regression model, where all the score components were included as independent covariates. Here, the diagnosis age was modeled with a spline with three degrees of freedom, grade (1,2,3) and the number of affected lymph nodes as numerical variables, tumor size (mm) as a log-scale linear variable, and PgR and Her2 statuses as dichotomous variables (positive vs. negative status). If any of the covariates was associated with significant residual hazard, a reduced score, excluding these covariates, was calculated. The reduced score was then used as an offset, and the hazard associated with these covariates was estimated with a multivariable Cox regression. All Cox regression models were stratified by country, to account for differences in the baseline risk due to differences in treatment practices. The offset models were further adjusted for diagnosis year (linear, continuous), to account for any residual improvement in therapy over the years.

The PREDICT calibration was studied separately in CIMBA and BCAC studies as a merged cohorts. The calibration was assessed by splitting the patient data into four (primarily) or three (if low number of cases) risk categories based on PREDICT percentiles (0–20%ile, 20–50%ile, 50–80%ile, 80–100%ile or 0–30%ile, 30–70%ile, 70–100%ile) and plotting the expected breast cancer mortality against the observed breast cancer mortality in the quantiles. Optimally, the calibration should be examined by comparing the expected and observed number of events. However, in right-censored, left-truncated data this was not possible, and the observed mortality and the respective cumulative hazard of state-transition was retrieved from Kaplan–Meier survival estimator, within each pooled dataset, after which the point-estimates and standard errors were pooled according to Rubin’s rules. The expected mortality was calculated separately in each imputed dataset based on the average PREDICT score and the baseline cumulative hazard of breast cancer death. The cumulative hazard estimates were pooled to average and transformed to expected mortality. The difference between expected and observed mortality was considered significant, if the expected mortality point-estimate was outside the pooled 95% confidence interval of the observed mortality. The breast cancer-specific survival of patients in the PREDICT risk categories was visualized with Kaplan–Meier graphs, separately in those CIMBA and BCAC studies, that provided cause of death information for at least 80% of deceased patients.

### Reporting summary

Further information on research design is available in the Nature Research Reporting Summary linked to this article.

## Supplementary information

Supplementary Information

Reporting Summary

## Data Availability

The consortia study participant phenotype data used in the current study are not publicly available due to protection of participant privacy and confidentiality, and data ownership belonging to the contributing institutions. But data can be made available in an anonymized form from the CIMBA and BCAC consortia on a reasonable request and after approval from the contributing studies. Requests for data can be made to the CIMBA and BCAC Data Access Coordination Committees (DACC; https://cimba.ccge.medschl.cam.ac.uk/projects/data-access-requests/; https://bcac.ccge.medschl.cam.ac.uk/bcacdata/). The contact person for data access requests is Manjeet Bolla (mkh39@medschl.cam.ac.uk) Data Manager, Department of Public Health and Primary Care, University of Cambridge. The imputed datasets are available from the corresponding author upon the DACC approval.

## Code availability

All statistical analyses were performed within the R environment for statistical computing version 4.0.0, including libraries *mice*, *survival*, and *CPE*. Custom code, used for pooling the imputed results, calculating the PREDICT scores and baseline risk is available in GitHub (https://github.com/TaruMuranen/PREDICT_for_BRCA1-2).
