# Supplementary material for: PREDICT validity for prognosis of breast cancer patients with pathogenic BRCA1/2 variants
Source: NPJ Breast Cancer. 2023 May 12;9:37. doi: 10.1038/s41523-023-00546-x (PMC10182045; doi:10.1038/s41523-023-00546-x)
Supplement: Supplementary file 1 — Supplementary Information [file 41523_2023_546_MOESM1_ESM.pdf]

PREDICT validity for prognosis of breast cancer patients with pathogenic *BRCA1/2* variants

Supplementary Note 1.

Acknowledgements

We thank all the individuals who took part in these studies and all the researchers, clinicians, technicians and administrative staff who have enabled this work to be carried out.

ABCS thanks the Blood bank Sanquin, The Netherlands. The ABCS study was supported by the Dutch Cancer Society [grants NKI 2007-3839; 2009 4363] and an institutional grant of the Dutch Cancer Society and of the Dutch Ministry of Health, Welfare and Sport.

The BREOGAN study would not have been possible without the contributions of the following: Manuela Gago-Dominguez, Jose Esteban Castelao, Angel Carracedo, Victor Muñoz Garzón, Alejandro Novo Domínguez, Maria Elena Martinez, Sara Miranda Ponte, Carmen Redondo Marey, Maite Peña Fernández, Manuel Enguix Castelo, Maria Torres, Manuel Calaza (BREOGAN), José Antúnez, Máximo Fraga and the staff of the Department of Pathology and Biobank of the University Hospital Complex of Santiago-CHUS, Instituto de Investigación Sanitaria de Santiago, IDIS, Xerencia de Xestion Integrada de Santiago-SERGAS; Joaquín González-Carreró and the staff of the Department of Pathology and Biobank of University Hospital Complex of Vigo, Instituto de Investigacion Biomedica Galicia Sur, SERGAS, Vigo, Spain. The BREast Oncology GAlician Network (BREOGAN) is funded by Acción Estratégica de Salud del Instituto de Salud Carlos III FIS PI12/02125/Cofinanciado and FEDER PI17/00918/Cofinanciado FEDER; Acción Estratégica de Salud del Instituto de Salud Carlos III FIS Intrasalud (PI13/01136); Programa Grupos Emergentes, Cancer Genetics Unit, Instituto de Investigacion Biomedica Galicia Sur. Xerencia de Xestion Integrada de Vigo-SERGAS, Instituto de Salud Carlos III, Spain; Grant 10CSA012E, Consellería de Industria Programa Sectorial de Investigación Aplicada, PEME I + D e I + D Suma del Plan Gallego de Investigación, Desarrollo e Innovación Tecnológica de la Consellería de Industria de la Xunta de Galicia, Spain; GranC

The BSUCH study acknowledges the Principal Investigator, Barbara Burwinkel, and, thanks Peter Bugert, Medical Faculty Mannheim.

CBCS thanks study participants, co-investigators, collaborators and staff of the Canadian Breast Cancer Study, and project coordinators Agnes Lai and Celine Morissette.

CCGP thanks Styliani Apostolaki, Anna Margiolaki, Georgios Nintos, Maria Perraki, Georgia Saloustrou, Georgia Sevastaki, Konstantinos Pompodakis.

CGPS thanks staff and participants of the Copenhagen General Population Study. For the excellent technical assistance: Dorthe Uldall Andersen, Maria Birna Arnadottir, Anne Bank, Dorthe Kjeldgård Hansen. The Danish Cancer Biobank is acknowledged for providing infrastructure for the collection of blood samples for the cases.

DFCI study is lead by Judy Garber.

The GENICA Network: Dr. Margarete Fischer-Bosch-Institute of Clinical Pharmacology, Stuttgart, and University of Tübingen, Germany [Hiltrud Brauch, Reiner Hoppe, Wing-Yee Lo], Department of Internal Medicine, Johanniter GmbH Bonn, Johanniter Krankenhaus, Bonn, Germany [YDK, Christian Baisch], Institute of Pathology, University of Bonn, Germany [Hans-Peter Fischer], Molecular Genetics of Breast Cancer, Deutsches Krebsforschungszentrum (DKFZ), Heidelberg, Germany [UH], Institute for Prevention and Occupational Medicine of the German Social Accident Insurance, Institute of the Ruhr University Bochum (IPA), Bochum, Germany [Thomas Brüning, Beate Pesch, Sylvia Rabstein, Anne Lotz]; and Institute of Occupational Medicine and Maritime Medicine, University Medical Center Hamburg-Eppendorf, Germany [Volker Harth].

HEBCS thanks Johanna Kiiski, Kirsimari Aaltonen, Karl von Smitten, Irja Erkkilä.

KARMA and SASBAC thank the Swedish Medical Research Counsel.

KBCP thanks Eija Myöhänen.

kConFab/AOCS wish to thank Heather Thorne, Eveline Niedermayr, all the kConFab research nurses and staff, the heads and staff of the Family Cancer Clinics, and the Clinical Follow Up Study (which has received funding from the NHMRC, the National Breast Cancer Foundation, Cancer Australia, and the National Institute of Health (USA)) for their contributions to this resource, and the many families who contribute to kConFab.

MARIE thanks Petra Seibold, Nadia Obi, Sabine Behrens, Ursula Eilber and Muhabbet Celik. MASTOS thanks all the study participants and express appreciation to the doctors: Yiola Marcou, Eleni Kakouri, Panayiotis Papadopoulos, Simon Malas and Maria Daniel, as well as to all the nurses and volunteers who provided valuable help towards the recruitment of the study participants.

MBCSG (Milan Breast Cancer Study Group): Paolo Radice, Siranoush Manoukian, Bernard Peissel, Jacopo Azzollini, Erica Rosina, Daniela Zaffaroni, Bernardo Bonanni, Irene Feroce, Mariarosaria Calvello, Aliana Guerrieri Gonzaga, Monica Marabelli, Davide Bondavalli and the personnel of the Cogentech Cancer Genetic Test Laboratory.

The MCCS was made possible by the contribution of many people, including the original investigators, the teams that recruited the participants and continue working on follow-up, and the many thousands of Melbourne residents who continue to participate in the study.

The following are NBCS Collaborators: Kristine K. Sahlberg (PhD), Anne-Lise Børresen-Dale (Prof. Em.), Lars Ottestad (MD), Rolf Kåresen (Prof. Em.) Dr. Ellen Schlichting (MD), Marit Muri Holmen (MD), Toril Sauer (MD), Vilde Haakensen (MD), Olav Engebråten (MD), Bjørn Naume (MD), Alexander Fosså (MD), Cecile E. Kiserud (MD), Kristin V. Reinertsen (MD), Åslaug Helland (MD), Margit Riis (MD), Jürgen Geisler (MD), OSBREAC and Grethe I. Grenaker Alnæs (MSc).

BCFR-ON/OCGN/OFBCR thank Teresa Selander, Nayana Weerasooriya and Steve Gallinger.

The Ontario Familial Breast Cancer Registry was supported by grant U01CA164920 from the USA National Cancer Institute of the National Institutes of Health. The content of this manuscript does not necessarily reflect the views or policies of the National Cancer Institute or any of the collaborating centers in the Breast Cancer Family Registry (BCFR), nor does mention of trade names, commercial products, or organizations imply endorsement by the USA Government or the BCFR.

PBCS thanks Louise Brinton, Mark Sherman, Neonila Szeszenia-Dabrowska, Beata Peplonska, Witold Zatonski, Pei Chao, Michael Stagner.

The RBCS thanks Jannet Blom, Saskia Pelders, Wendy J.C. Prager – van der Smissen, and the Erasmus MC Family Cancer Clinic. SBCS thanks Sue Higham, Helen Cramp, Dan Connley, Ian Brock, Sabapathy Balasubramanian and Malcolm W.R. Reed.

We thank the SEARCH team.

SKKDKFZS thanks all study participants, clinicians, family doctors, researchers and technicians for their contributions and commitment to this study.

SZBCS and IHCC were supported by PBZ_KBN_122/P05/2004 and the program of the Minister of Science and Higher Education under the name "Regional Initiative of Excellence" in 2019–2022 project number 002/RID /2018/19 amount of financing12 000 000 PLN.

Swedish Breast Cancer Study (SWE-BRCA) consists of the following collaborating centers: Lund University, Lund, Sweden: Åke Borg, Håkan Olsson, Helena Jernström, Karin Henriksson, Katja Harbst, Maria Soller, Ulf Kristoffersson; Gothenburg Sahlgrenska University Hospital, Gothenburg, Sweden: Anna Öfverholm, Margareta Nordling, Per Karlsson, Zakaria Einbeigi; Karolinska University Hospital, Stockholm, Sweden: Anna von Wachenfeldt, Annelie Liljegren, Annika Lindblom, Brita Arver, Gisela Barbany Bustinza; Umeå University Hospital, Umeå, Sweden: Beatrice Melin, Christina Edwinsdotter Ardnor, Monica Emanuelsson; Uppsala University, Uppsala, Sweden: Hans Ehrencrona, Maritta Hellström Pigg, Richard Rosenquist; Linköping University Hospital, Linköping, Sweden: Marie Stenmark-Askmalm, Sigrun Liedgren.

UBCS thanks all study participants as well as the ascertainment, laboratory, analytics and informatics teams at Huntsman Cancer Institute and Intermountain Healthcare for their important contributions to this study.

UPENN: Breast Cancer Research Foundation; Susan G. Komen Foundation for the cure, Basser Research Center for BRCA.

Supplementary Note 2.

HEBON investigators

The Hereditary Breast and Ovarian Cancer Research Group Netherlands (**HEBON**) consists of the following collaborating centers: Netherlands Cancer Institute (coordinating center), Amsterdam, NL: M.A. Rookus, F.B.L. Hogervorst, F.E. van Leeuwen, M.A. Adank, D.J. Jenner; Erasmus Medical Center, Rotterdam, NL: J.M. Collée, A.M.W. van den Ouweland, I.A. Boere; Leiden University Medical Center, NL: C.J. van Asperen, P. Devilee, R.B. van der Luijt, T.C.T.E.F. van Cronenburg; Radboud University Nijmegen Medical Center, NL: M.R. Wevers, A.R. Mensenkamp; University Medical Center Utrecht, NL: M.G.E.M. Ausems, M.J. Koudijs; Amsterdam Medical Center, NL: T.A.M. van Os; VU University Medical Center, Amsterdam, NL: K. van Engelen, J.J.P. Gille; Maastricht University Medical Center, NL: E.B. Gómez-Garcia, M.J. Blok, M. de Boer; University of Groningen, NL: L.P.V. Berger, A.H. van der Hout, M.J.E. Mourits, G.H. de Bock; The Netherlands Comprehensive Cancer Organisation (IKNL): S. Siesling, J. Verloop; The nationwide network and registry of histo- and cytopathology in The Netherlands (PALGA): E.C. van den Broek.

Supplementary table 1. Predictor matrix for multiple imputation.

		Study‡	BRCA1/2	Age	Year	Entry	Vital status	Cum. Haz. Overall	Cum. Haz. BC	ER	PgR	Her2	Grade	Size	Nodecount	Endocrine therapy	Anthracyclines	Taxanes	CMF	Trastuzumab	BC death	BC death or 2nd BC	Stage	Family history	N	Morphology	Size category	Metastasis	Chemotherapy	Tamoxifen	Aromatase inhibitor	Surgery	Radiation	Neoadjuvant chemotherapy	Anthracyclines neoadjuvan	Taxanes neoadjuvant	CMF neoadjuvant	ID	Follow-up time	Vitalstatus or 2nd BC	Follow-up cens. 2nd BC	Consortium	Country-stratum
Predictive features with no missing values	Study group (group name: categorical)†	0	0	0	0	0	0	0	0	0	0	0	0	0	0	0	0	0	0	0	0	0	0	0	0	0	0	0	0	0	0	0	0	0	0	0	0	0	0	0	0	0	0
	Germline BRCA1/2 pathogenic variant (BRCA1, BRCA2, VUS, no: categorical)	0	0	0	0	0	0	0	0	0	0	0	0	0	0	0	0	0	0	0	0	0	0	0	0	0	0	0	0	0	0	0	0	0	0	0	0	0	0	0	0	0	0
	Diagnosis age (age: numeric)	0	0	0	0	0	0	0	0	0	0	0	0	0	0	0	0	0	0	0	0	0	0	0	0	0	0	0	0	0	0	0	0	0	0	0	0	0	0	0	0	0	0
	Diagnosis year (year: numeric)	0	0	0	0	0	0	0	0	0	0	0	0	0	0	0	0	0	0	0	0	0	0	0	0	0	0	0	0	0	0	0	0	0	0	0	0	0	0	0	0	0	0
Predictive features, only observed values used in the analyses	Time from diagnosis to study entry (years: numeric)	**1**	**1**	**1**	**1**	0	0	**1**	**1**	0	0	0	0	0	0	**1**	**1**	0	0	0	0	0	0	**1**	0	0	0	0	0	0	0	0	0	0	0	0	0	0	0	0	0	0	0
	Vital status at the end of follow-up (alive/dead: dichotomous)	**1**	**1**	0	**1**	0	0	**1**	**1**	0	0	0	0	0	**1**	**1**	0	0	0	0	0	0	0	**1**	0	0	**1**	**1**	0	0	0	0	0	0	0	0	0	0	0	0	0	0	0
	Nelson-Aalen cumulative hazard estimate for all-cause death at the end of the follow-up (probal	**1**	**1**	0	**1**	**1**	**1**	0	**1**	0	0	0	**1**	0	0	0	**1**	**1**	0	0	**1**	0	0	**1**	0	0	0	0	0	0	0	0	0	**1**	0	**1**	0	0	0	0	0	0	0
	Nelson-Aalen cumulative hazard estimate for breast cancer-associated death at the end of the	**1**	**1**	0	**1**	**1**	**1**	**1**	0	0	0	0	**1**	0	0	0	**1**	**1**	0	0	**1**	0	0	0	0	0	0	**1**	**1**	0	0	0	0	**1**	0	0	0	0	0	0	0	0	0
Predictive features with missing values, included in the analyses	Tumor ER-status (negative, positive: dichotomous)	**1**	**1**	**1**	0	**1**	0	**1**	**1**	0	**1**	0	**1**	0	0	**1**	**1**	0	**1**	0	0	0	0	**1**	0	**1**	0	0	**1**	**1**	**1**	0	0	0	0	0	0	0	0	0	0	0	0
	Tumor PgR-status (negative, positive: dichotomous)	**1**	**1**	0	0	**1**	0	**1**	**1**	**1**	0	0	**1**	0	0	**1**	**1**	**1**	0	0	0	0	0	0	0	0	0	0	**1**	**1**	**1**	0	0	0	0	0	0	0	0	0	0	0	0
	Tumor Her2-status (negative, positive: dichotomous)	**1**	0	0	0	0	0	**1**	**1**	0	**1**	0	**1**	0	0	0	**1**	**1**	0	**1**	0	0	0	0	0	0	0	0	**1**	0	0	0	0	0	0	0	0	0	0	0	0	0	0
	Tumor grade (1,2,3: categorical)	**1**	**1**	**1**	0	**1**	0	**1**	**1**	**1**	**1**	**1**	0	0	**1**	**1**	**1**	**1**	**1**	0	0	0	0	**1**	0	**1**	**1**	0	**1**	**1**	0	**1**	**1**	0	0	0	0	0	0	0	0	0	0
	Tumor size (maximum diameter in mm on a log-scale: numeric)	0	0	0	0	0	0	0	0	0	0	0	0	0	0	0	0	0	0	0	0	0	0	0	0	0	**1**	0	0	0	0	0	0	0	0	0	0	0	0	0	0	0	0
	Number of affected lymph nodes (count: numeric)	**1**	**1**	0	0	0	**1**	**1**	**1**	0	0	0	**1**	0	0	0	**1**	**1**	0	0	**1**	0	0	0	**1**	0	**1**	0	**1**	0	0	**1**	**1**	**1**	0	0	0	0	0	0	0	0	0
	Any adjuvant endocrine therapy (no, yes: dichotomous)	**1**	**1**	**1**	**1**	**1**	0	**1**	**1**	**1**	**1**	0	**1**	0	0	0	0	0	0	0	0	0	0	**1**	0	0	0	0	0	0	0	0	0	0	0	0	0	0	0	0	0	0	0
	Any anthracycline-based adjuvant chemotherapy (no, yes: dichotomous)	**1**	0	**1**	**1**	0	0	0	0	0	0	0	0	0	0	0	0	0	0	0	0	0	0	0	0	0	0	0	**1**	0	0	0	0	0	0	0	0	0	0	0	0	0	0
	Any taxane-based adjuvant chemotherapy (no, yes: dichotomous)	**1**	0	**1**	**1**	0	0	0	0	0	0	0	0	0	0	0	0	0	0	0	0	0	0	0	0	0	0	0	**1**	0	0	0	0	0	0	0	0	0	0	0	0	0	0
	Any CMF-based adjuvant chemotherapy (no, yes: dichotomous)	**1**	0	**1**	**1**	0	0	0	0	0	0	0	0	0	0	0	0	0	0	0	0	0	0	0	0	0	0	0	**1**	0	0	0	0	0	0	0	0	0	0	0	0	0	0
	Adjuvant trastuzumab therapy (no, yes: dichotomous)	**1**	0	0	**1**	0	0	0	0	0	0	**1**	0	0	0	0	0	0	0	0	0	0	0	0	0	0	0	0	0	0	0	0	0	0	0	0	0	0	0	0	0	0	0
	Breast cancer-associated death (alive, dead from BC, dead from other cause: categorical)	**1**	**1**	0	**1**	0	**1**	**1**	**1**	0	0	0	0	0	**1**	**1**	0	0	0	0	0	0	0	**1**	0	0	**1**	**1**	0	0	0	0	0	0	0	0	0	0	0	0	0	0	0
Non-predictive features, whose values were defined by decision rules	Breast cancer associated death or second breast cancer (as above or second BC: categorical)	0	0	0	0	0	0	0	0	0	0	0	0	0	0	0	0	0	0	0	0	0	0	0	0	0	0	0	0	0	0	0	0	0	0	0	0	0	0	0	0	0	0
	Tumor stage (xxx: categorical)	0	0	0	0	0	0	0	0	0	0	0	0	0	0	0	0	0	0	0	0	0	0	0	0	0	0	0	0	0	0	0	0	0	0	0	0	0	0	0	0	0	0
Predictive features with missing data, not included in the analyses	Family history of BC (no, yes: categorical)	**1**	**1**	**1**	0	**1**	0	**1**	**1**	**1**	0	0	**1**	0	0	**1**	0	0	**1**	0	0	0	0	0	0	0	0	0	0	0	0	0	**1**	0	0	0	0	0	0	0	0	0	0
	Lymph node status (negative, positive: dichotomous)	**1**	0	0	0	0	**1**	**1**	**1**	0	0	0	0	0	0	0	**1**	**1**	**1**	0	**1**	0	0	0	0	0	**1**	0	**1**	0	0	**1**	**1**	**1**	0	0	0	0	0	0	0	0	0
	Tumor morphology (morphology: categorical)	**1**	**1**	0	0	**1**	0	**1**	**1**	**1**	0	0	**1**	0	0	0	0	0	0	0	0	0	0	**1**	0	0	0	0	0	0	0	0	0	0	0	0	0	0	0	0	0	0	0
	Tumor size category (1, 2, 3: categorical)	**1**	**1**	**1**	0	0	**1**	**1**	**1**	0	0	0	**1**	0	**1**	0	**1**	0	0	0	**1**	0	0	**1**	0	0	0	0	**1**	0	0	**1**	0	**1**	**1**	0	**1**	0	0	0	0	0	0
	Distant metastasis at diagnosis (no, yes: dichotomous)	**1**	**1**	**1**	0	**1**	**1**	**1**	**1**	0	0	0	0	0	**1**	**1**	**1**	0	0	**1**	**1**	0	0	**1**	0	0	**1**	0	0	0	**1**	0	0	0	0	0	0	0	0	0	0	0	0
	Any adjuvant chemotherapy (no, yes: dichotomous)	**1**	**1**	**1**	**1**	**1**	0	**1**	**1**	**1**	**1**	0	**1**	0	**1**	0	0	0	0	0	0	0	0	**1**	0	0	**1**	0	0	0	0	0	0	0	0	0	0	0	0	0	0	0	0
	Adjuvant tamoxifen therapy (no, yes: dichotomous)	**1**	0	**1**	**1**	0	0	0	0	0	0	0	0	0	0	**1**	0	0	0	0	0	0	0	0	0	0	0	0	0	0	0	0	0	0	0	0	0	0	0	0	0	0	0
	Adjuvant aromatase inhibitor therapy (no, yes: dichotomous)	**1**	0	**1**	**1**	0	0	0	0	0	0	0	0	0	0	**1**	0	0	0	0	0	0	0	0	0	0	0	0	0	0	0	0	0	0	0	0	0	0	0	0	0	0	0
	Surgery type (breast conserving, mastectomy)	**1**	0	0	**1**	0	0	**1**	**1**	0	0	0	**1**	0	**1**	**1**	**1**	**1**	**1**	0	0	0	0	0	0	0	**1**	0	**1**	**1**	0	0	**1**	0	0	0	0	0	0	0	0	0	0
	Adjuvant radiation therapy (no, yes: dichotomous)	**1**	**1**	**1**	**1**	**1**	0	**1**	**1**	0	0	0	0	0	**1**	0	0	0	0	0	0	0	0	**1**	0	0	0	0	0	0	0	0	0	0	0	0	0	0	0	0	0	0	0
	Any neoadjuvant chemotherapy (no, yes: dichotomous)	**1**	0	**1**	**1**	**1**	0	**1**	**1**	0	0	0	0	0	**1**	**1**	0	0	0	0	0	0	0	**1**	0	0	**1**	0	0	0	0	0	0	0	0	0	0	0	0	0	0	0	0
	Any anthracycline-based neoadjuvant chemotherapy (no, yes: dichotomous)	**1**	0	**1**	**1**	0	0	0	0	0	0	0	0	0	0	0	0	0	0	0	0	0	0	0	0	0	0	0	0	0	0	0	0	**1**	0	0	0	0	0	0	0	0	0
	Any taxane-based adjuvant neochemotherapy (no, yes: dichotomous)	**1**	0	**1**	**1**	0	0	0	0	0	0	0	0	0	0	0	0	0	0	0	0	0	0	0	0	0	0	0	0	0	0	0	0	**1**	0	0	0	0	0	0	0	0	0
	Any CMF-based adjuvant neochemotherapy (no, yes: dichotomous)	**1**	0	**1**	**1**	0	0	0	0	0	0	0	0	0	0	0	0	0	0	0	0	0	0	0	0	0	0	0	0	0	0	0	0	**1**	0	0	0	0	0	0	0	0	0
Features excluded from imputation (non-predictive features)	Patient ID	0	0	0	0	0	0	0	0	0	0	0	0	0	0	0	0	0	0	0	0	0	0	0	0	0	0	0	0	0	0	0	0	0	0	0	0	0	0	0	0	0	0
	Follow-up time	0	0	0	0	0	0	0	0	0	0	0	0	0	0	0	0	0	0	0	0	0	0	0	0	0	0	0	0	0	0	0	0	0	0	0	0	0	0	0	0	0	0
	Alive, dead or second breast cancer	0	0	0	0	0	0	0	0	0	0	0	0	0	0	0	0	0	0	0	0	0	0	0	0	0	0	0	0	0	0	0	0	0	0	0	0	0	0	0	0	0	0
	Follow-up time censored at occurrence of second breast cancer	0	0	0	0	0	0	0	0	0	0	0	0	0	0	0	0	0	0	0	0	0	0	0	0	0	0	0	0	0	0	0	0	0	0	0	0	0	0	0	0	0	0
	Concortium (CIMBA, BCAC)	0	0	0	0	0	0	0	0	0	0	0	0	0	0	0	0	0	0	0	0	0	0	0	0	0	0	0	0	0	0	0	0	0	0	0	0	0	0	0	0	0	0
	Country-stratum	0	0	0	0	0	0	0	0	0	0	0	0	0	0	0	0	0	0	0	0	0	0	0	0	0	0	0	0	0	0	0	0	0	0	0	0	0	0	0	0	0	0

† Each row lists the predictors of the feature named in this column.
‡ Each column lists all features predicted by the feature named on this row.
The order of the features is the same in rows and columns, the column feature names are abreviated.

Supplementary table 2. Residual hazard associated with PREDICT covariates

All covariates were refitted in a country-stratified multivariable Cox regression model, offsetting with the ER-negative PREDICT score. The model was fitted to data from CIMBA *BRCA1* carriers with ER-negative breast cancer.

**Refitted factors (m=50)**	**HR**	**95% confidence interval**	**P**
Diagnosis age (years)	1.01	0.99-1.02	0.50
Diagnosis year (1990-2011)	0.98	0.94-1.01	0.29
Tumor grade	0.67	0.43-1.03	0.16
Progesterone receptor expression	1.61	0.75-3.47	0.32
HER2 expression	0.69	0.37-1.26	0.41
Tumor size (mm, log-transformed)	1.15	0.88-1.50	0.50
Positive node count	1.02	1.00-1.05	0.18

Supplementary Table 3. Hazard associated with tumor grade

The country-stratified multivariable Cox regression model, was offset with a reduced score including coefficients and functions for tumor size, number of affected nodes, and diagnosis age as implemented in the ER-positive PREDICT score. The model was fitted to data from CIMBA BRCA2 carriers with ER-positive breast cancer.

(m=50)	**HR**	**95% confidence interval**	**P**
**Grade1 vs grade2**	0.89	0.41-1.92	0.83
**Grade3 vs grade2**	0.68	0.48-0.96	0.072
**Diagnosis year**	0.98	0.94-1.03	0.50

Supplementary Table 4. Reported adjuvant therapy regimens for BRCA2 carriers with ER-positive breast cancer

	Grade 1	Grade 2	Grade 3	Stratified Cochran-Mantel-Haenszel test
**No adjuvant therapy**	3<br>*12%*	10<br>*5%*	10<br>*5%*	Grade 3 vs. Grade 2<br>P: 0.0017
**Endocrine therapy only**	18<br>*72%*	74<br>*36%*	48<br>*22%*	
**Chemotherapy only**	1<br>*4%*	16<br>*8%*	23<br>*11%*	
**Chemotherapy with endocrine therapy**	3<br>*12%*	107<br>*52%*	135<br>*62%*	

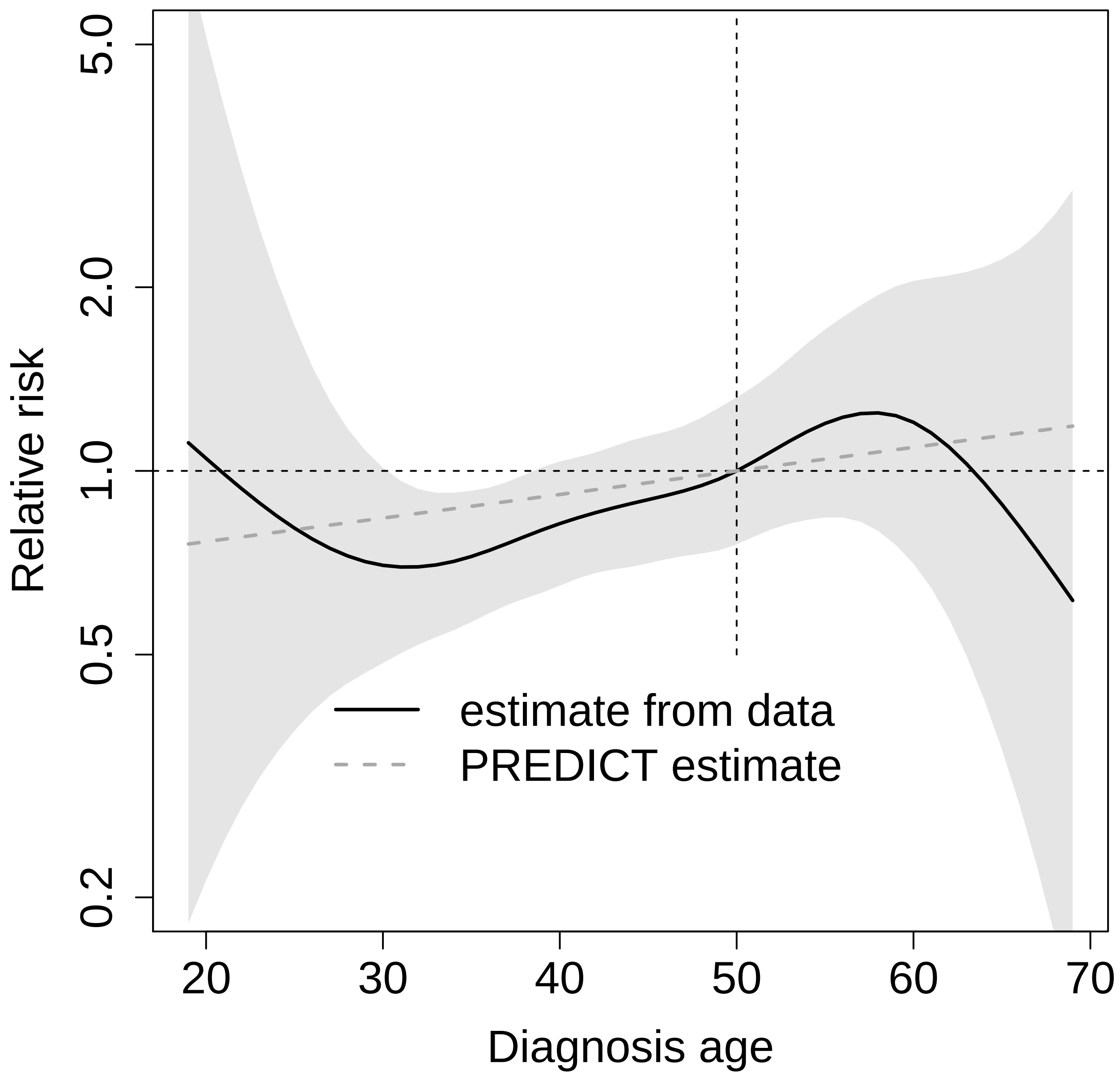

Supplementary Figure 1. *BRCA1* carriers' age-related breast cancer mortality
The relative risk associated with diagnosis age was estimated in reference to a patient diagnosed with breast cancer at the age of 50 years, using data from CIMBA *BRCA1* carrier patients with ER-negative breast cancer. (Shaded area indicates the 95% confidence interval of the age-related risk estimate from the data.)

a

CIMBA ER− *BRCA1*, 5−year risk of BC−death

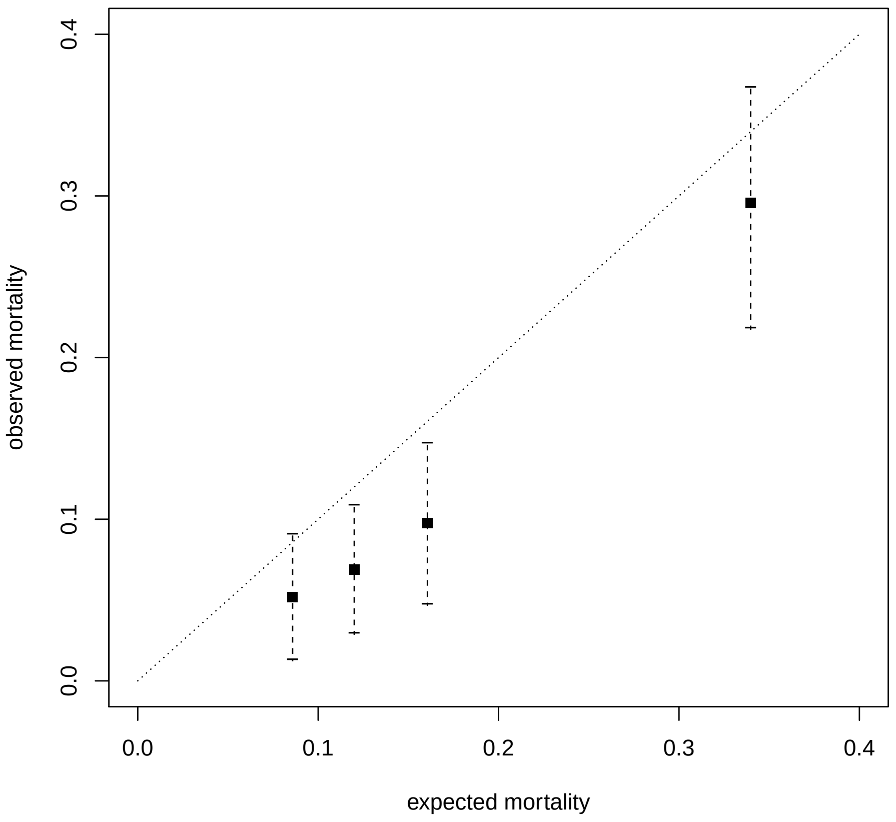

b

CIMBA ER− *BRCA1*, 15−year risk of BC−death

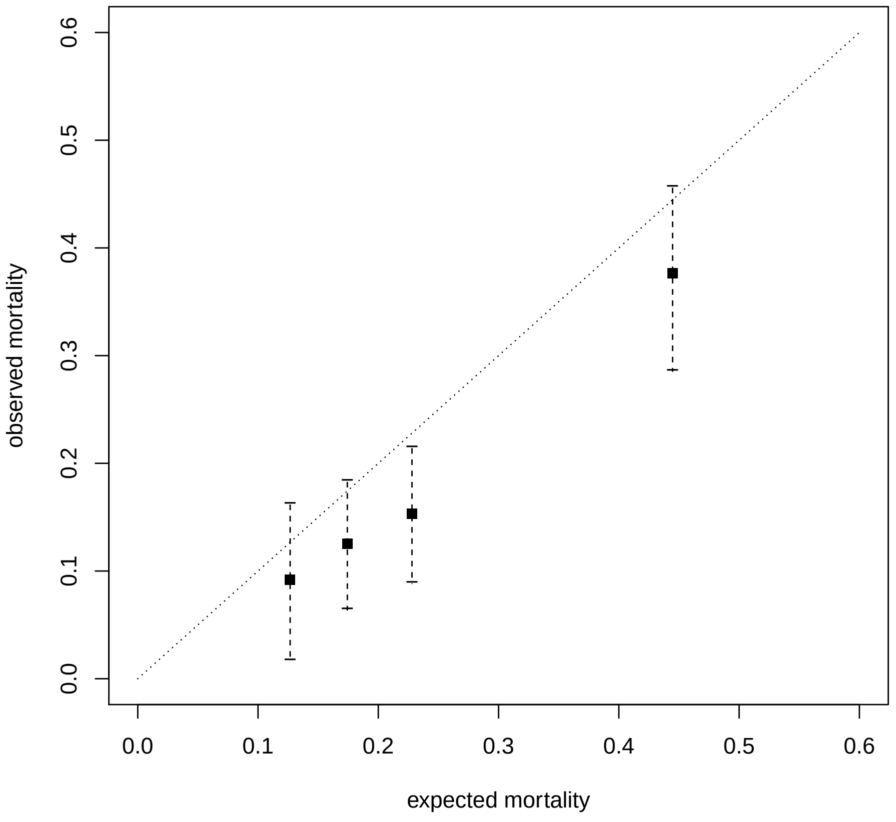

Supplementary Figure 2. ER-negative PREDICT calibration

The expected breast cancer mortality plotted against the observed breast cancer mortality (pooled Nelson-Ahlen mortality estimate of mortality with 95% confidence interval) in different risk categories, defined by the PREDICT percentiles: 0-20%ile, 20-50%ile, 50-80%ile, 80-100%ile (from left to right in every plot). ER-negative PREDICT calibration in *BRCA1* variant carriers from merged CIMBA studies for a) 5- and b) 15-year follow-up time.

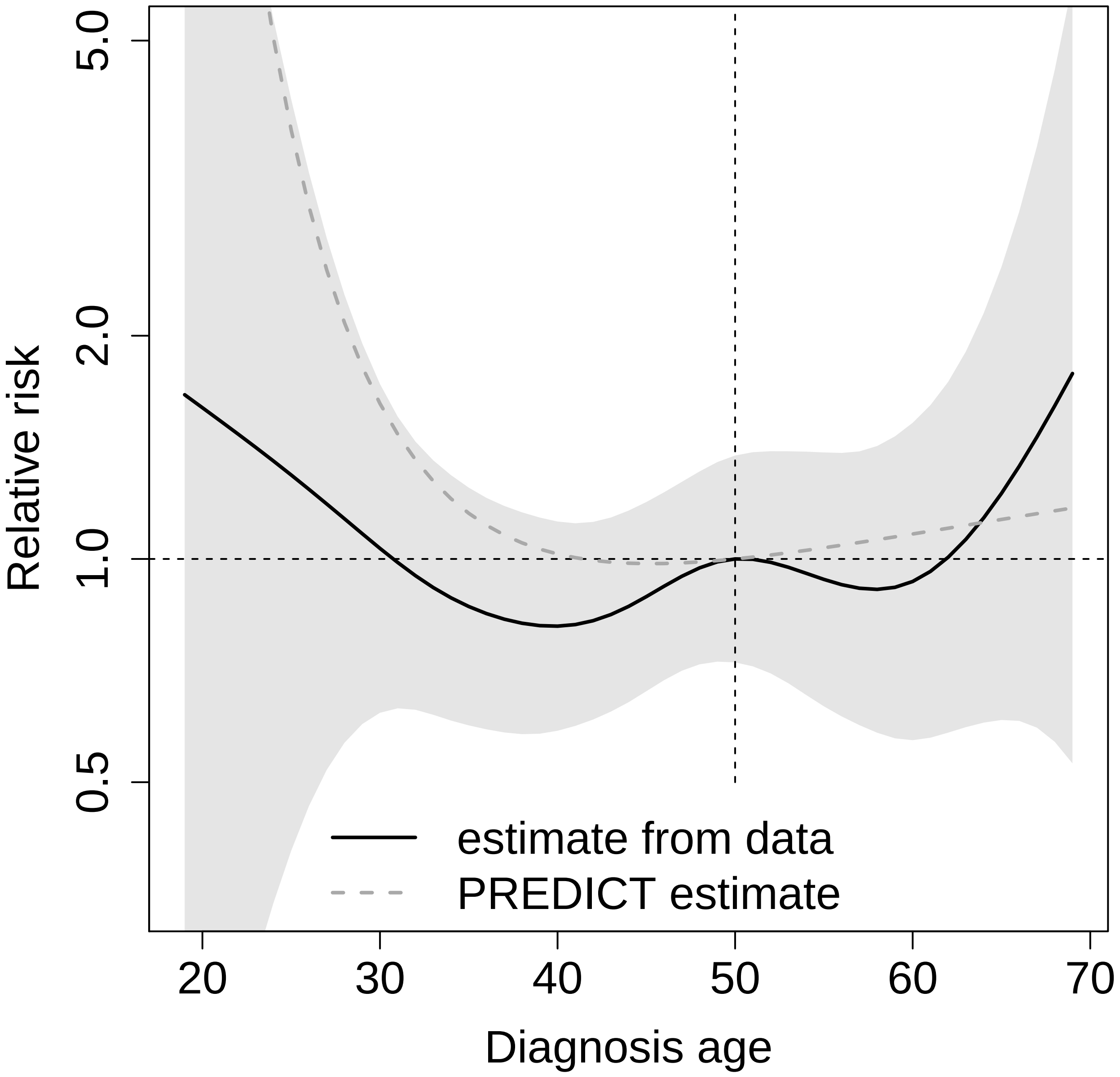

Supplementary Figure 3. *BRCA2* carriers' age-related breast cancer mortality

The relative risk associated with diagnosis age was estimated in reference to a patient diagnosed with breast cancer at the age of 50 years, using data from CIMBA *BRCA2* carrier patients with ER-positive breast cancer. (Shaded area indicates the 95% confidence interval of the age-related risk estimate from the data.)

a

CIMBA ER+ *BRCA2*, 5−year risk of BC−death

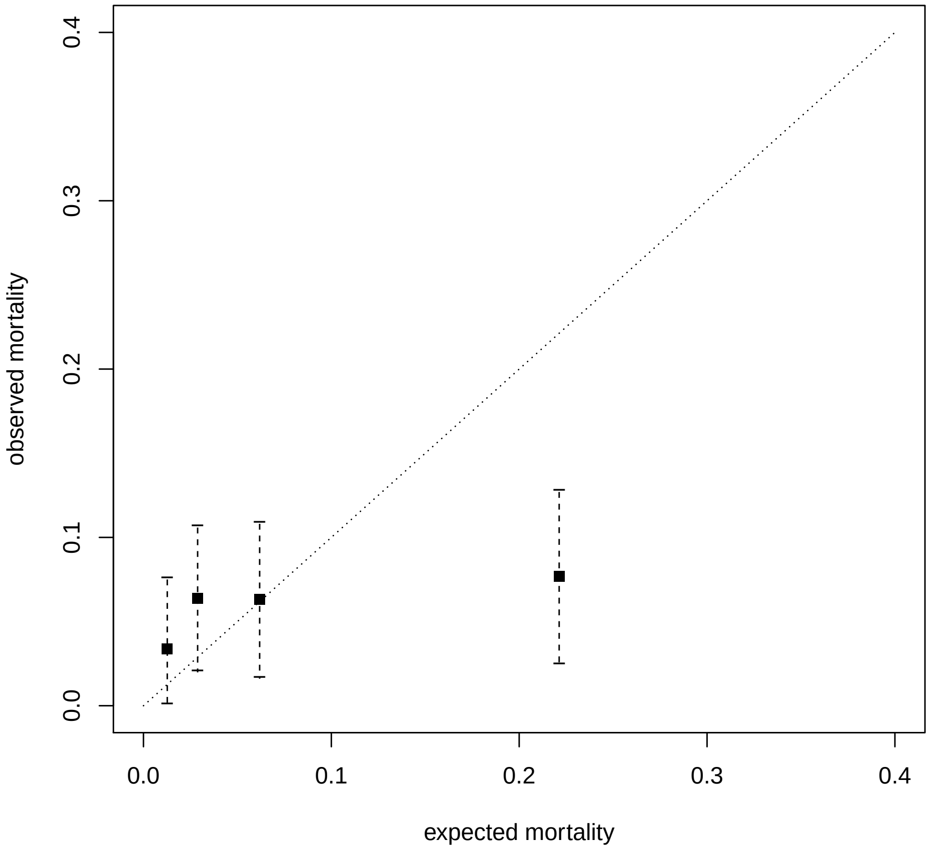

b

CIMBA ER+ *BRCA2*, 15−year risk of BC−death

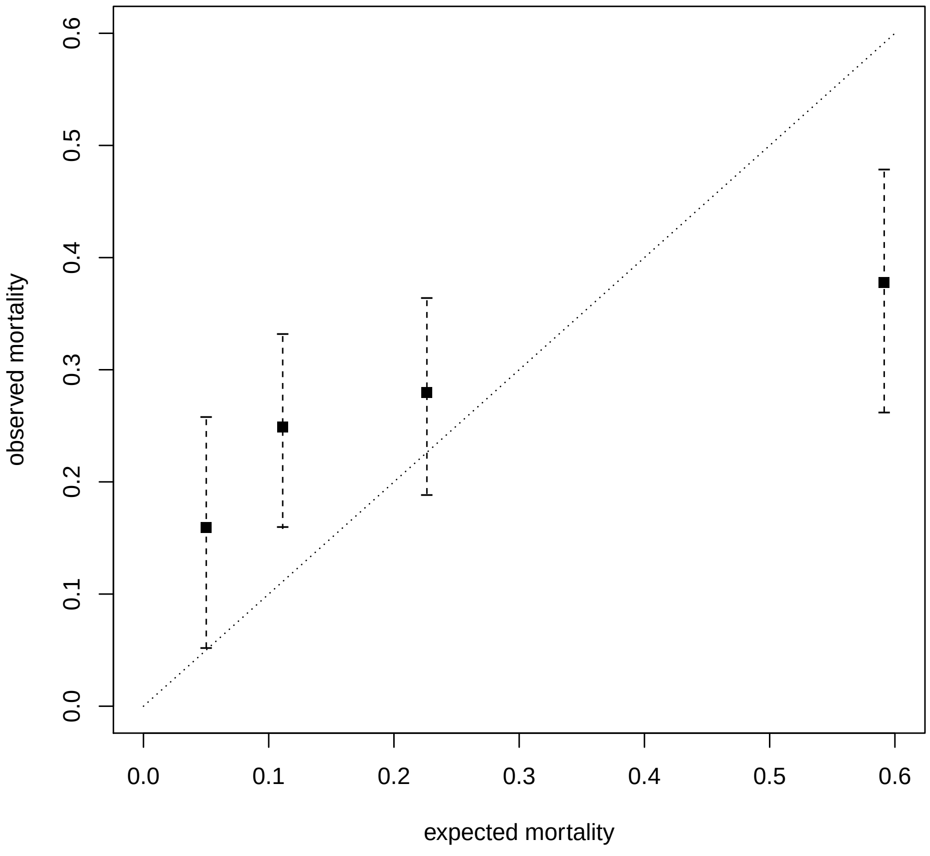

Supplementary Figure 4. ER-positive PREDICT calibration

The expected breast cancer mortality plotted against the observed breast cancer mortality (pooled Nelson-Ahlen mortality estimate of mortality with 95% confidence interval) in different risk categories, defined by the PREDICT per-centiles: 0-20%ile, 20-50%ile, 50-80%ile, 80-100%ile (from left to right in every plot). ER-positive PREDICT calibration in *BRCA2* variant carriers from merged CIMBA studies for a) 5- and b) 15-year follow-up time.
