# Supplementary material for: PREDICT validity for prognosis of breast cancer patients with pathogenic BRCA1/2 variants
Source: NPJ Breast Cancer. 2023 May 12;9:37. doi: 10.1038/s41523-023-00546-x (PMC10182045; doi:10.1038/s41523-023-00546-x)
Supplement: Supplementary file 2 — Reporting Summary [file 41523_2023_546_MOESM2_ESM.pdf]

nature portfolio

Corresponding author(s): Taru Muranen

Last updated by author(s): Nov 13, 2022

Reporting Summary

Nature Portfolio wishes to improve the reproducibility of the work that we publish. This form provides structure for consistency and transparency in reporting. For further information on Nature Portfolio policies, see our Editorial Policies and the Editorial Policy Checklist.

Statistics

For all statistical analyses, confirm that the following items are present in the figure legend, table legend, main text, or Methods section.

n/a	Confirmed	
☐	☒	The exact sample size (*n*) for each experimental group/condition, given as a discrete number and unit of measurement
☒	☐	A statement on whether measurements were taken from distinct samples or whether the same sample was measured repeatedly
☐	☒	The statistical test(s) used AND whether they are one- or two-sided<br>*Only common tests should be described solely by name; describe more complex techniques in the Methods section.*
☐	☒	A description of all covariates tested
☐	☒	A description of any assumptions or corrections, such as tests of normality and adjustment for multiple comparisons
☐	☒	A full description of the statistical parameters including central tendency (e.g. means) or other basic estimates (e.g. regression coefficient) AND variation (e.g. standard deviation) or associated estimates of uncertainty (e.g. confidence intervals)
☐	☒	For null hypothesis testing, the test statistic (e.g. *F*, *t*, *r*) with confidence intervals, effect sizes, degrees of freedom and *P* value noted<br>*Give P values as exact values whenever suitable.*
☒	☐	For Bayesian analysis, information on the choice of priors and Markov chain Monte Carlo settings
☒	☐	For hierarchical and complex designs, identification of the appropriate level for tests and full reporting of outcomes
☐	☒	Estimates of effect sizes (e.g. Cohen's *d*, Pearson's *r*), indicating how they were calculated

Our web collection on statistics for biologists contains articles on many of the points above.

Software and code

Policy information about availability of computer code

Data collection	R version 4.0.0, library mice
Data analysis	R version 4.0.0, libraries survival, mice, CPE<br>Custom code, used for pooling the imputed results, calculating the PREDICT scores and baseline risk will available in GitHub (https://github.com/TaruMuranen/PREDICT_for_BRCA1-2) upon manuscript publication.

For manuscripts utilizing custom algorithms or software that are central to the research but not yet described in published literature, software must be made available to editors and reviewers. We strongly encourage code deposition in a community repository (e.g. GitHub). See the Nature Portfolio guidelines for submitting code & software for further information.

Data

Policy information about availability of data

All manuscripts must include a data availability statement. This statement should provide the following information, where applicable:

- Accession codes, unique identifiers, or web links for publicly available datasets
- A description of any restrictions on data availability
- For clinical datasets or third party data, please ensure that the statement adheres to our policy

Requests for data can be made to the CIMBA or BCAC Data Access Coordination Committee. The contact persons for data access requests are Lesley McGuffog

(ljm26@medschl.cam.ac.uk), Data Manager, Department of Public Health and Primary Care, University of Cambridge and Manjeet Bolla (mkh39@medschl.cam.ac.uk) Data Manager, Department of Public Health and Primary Care, University of Cambridge.

Human research participants

Policy information about studies involving human research participants and Sex and Gender in Research.

Reporting on sex and gender	The studied phenotype is female breast cancer. All study participants were female.
Population characteristics	The study subjects included female breast cancer patients of European ethnic origin enrolled into studies participating in the CIMBA or BCAC. The inclusion criterion was germline pathogenic variant in BRCA1 or BRCA2 gene.
Recruitment	CIMBA study subjects were recruited mainly in family cancer clinics after detection of a pathogenic BRCA1/2 variant. BCAC included hospital and population based cohorts. However, only BRCA1/2 carrier were included from these studies.
Ethics oversight	All participating studies were approved by their appropriate ethics review boards, and followed their national guidelines for informed consent.

Note that full information on the approval of the study protocol must also be provided in the manuscript.

Field-specific reporting

Please select the one below that is the best fit for your research. If you are not sure, read the appropriate sections before making your selection.

☒ Life sciences ☐ Behavioural & social sciences ☐ Ecological, evolutionary & environmental sciences

For a reference copy of the document with all sections, see nature.com/documents/nr-reporting-summary-flat.pdf

Life sciences study design

All studies must disclose on these points even when the disclosure is negative.

Sample size	Sample size was the number of study subjects, 2892 BRCA1 carriers and 1813 BRCA2 carriers from CIMBA, and 316 BRCA1 carriers and 432 BRCA2 carriers from BCAC.
Data exclusions	Patients were required to have data on the time of study entry, the length of follow-up and vital status at the end of the follow-up.
Replication	CIMBA patients were the discovery cohort and findings were validated with the BCAC patients.
Randomization	Analysis groups were defined by the breast cancer pathologic characteristics and pathogenic variant status.
Blinding	Not applicable.

Reporting for specific materials, systems and methods

We require information from authors about some types of materials, experimental systems and methods used in many studies. Here, indicate whether each material, system or method listed is relevant to your study. If you are not sure if a list item applies to your research, read the appropriate section before selecting a response.

Materials & experimental systems

n/a	Involved in the study	
☒	☐	Antibodies
☒	☐	Eukaryotic cell lines
☒	☐	Palaeontology and archaeology
☒	☐	Animals and other organisms
☒	☐	Clinical data
☒	☐	Dual use research of concern

Methods

n/a	Involved in the study	
☒	☐	ChIP-seq
☒	☐	Flow cytometry
☒	☐	MRI-based neuroimaging
